# Exploring the relationship between governance mechanisms in healthcare and health workforce outcomes: a systematic review

**DOI:** 10.1186/1472-6963-14-479

**Published:** 2014-10-04

**Authors:** Stephanie E Hastings, Gail D Armitage, Sara Mallinson, Karen Jackson, Esther Suter

**Affiliations:** Alberta Health Services, 10301 Southport Lane SW, Calgary, AB T2W 1S7 Canada

**Keywords:** Workforce outcomes, Governance, Quality improvement, Work attitudes, Magnet accreditation, Shared governance, Healthcare delivery, Review

## Abstract

**Background:**

The objective of this systematic review of diverse evidence was to examine the relationship between health system governance and workforce outcomes. Particular attention was paid to how governance mechanisms facilitate change in the workforce to ensure the effective use of all health providers.

**Methods:**

In accordance with standard systematic review procedures, the research team independently screened over 4300 abstracts found in database searches, website searches, and bibliographies. Searches were limited to 2001–2012, included only publications from Canada, the United Kingdom, the Netherlands, New Zealand, Australia, and the United States. Peer- reviewed papers and grey literature were considered. Two reviewers independently rated articles on quality and relevance and classified them into themes identified by the team. One hundred and thirteen articles that discussed both workforce and governance were retained and extracted into narrative summary tables for synthesis.

**Results:**

Six types of governance mechanisms emerged from our analysis. *Shared governance*, *Magnet accreditation*, and *professional development initiatives* were all associated with improved outcomes for the health workforce (e.g., decreased turnover, increased job satisfaction, increased empowerment, etc.). Implementation of *quality-focused initiatives* was associated with apprehension among providers, but opportunities for provider training on these initiatives increased quality and improved work attitudes. Research on *reorganization of healthcare delivery* suggests that changing to team-based care is accompanied by stress and concerns about role clarity, that outcomes vary for providers in private versus public organizations, and that co-operative clinics are beneficial for physicians. *Funding schemes* required a supplementary search to achieve adequate depth and coverage. Those findings are reported elsewhere.

**Conclusions:**

The results of the review show that while there are governance mechanisms that consider workforce impacts, it is not to the extent one might expect given the importance of the workforce for improving patient outcomes. Furthermore, to successfully implement governance mechanisms in this domain, there are key strategies recommended to support change and achieve desired outcomes. The most important of these are: to build trust by clearly articulating the organization’s goal; considering the workforce through planning, implementation, and evaluation phases; and providing strong leadership.

**Electronic supplementary material:**

The online version of this article (doi:10.1186/1472-6963-14-479) contains supplementary material, which is available to authorized users.

## Background

Over the last decade, Canadian health systems have undergone numerous changes. The main drivers for change are issues of sustainability [1], perceived health human resource [HHR] shortages [2, 3] and the desire to improve health outcomes [2, 4]. Significant health system transformation involves changes in structure, processes, culture, and values [5] which better align care with population health needs.

A key element of system transformation is an educated and skilled workforce [1], with appropriate skill mix, that is utilized in an effective and efficient way [6]. Changing the way healthcare providers work together to deliver care may address potential HHR shortages, healthcare provider misdistribution, and help achieve high quality, efficient, and cost-effective care. Furthermore, workforce modification is expected to increase productivity, job satisfaction, recruitment, and retention [7], leading to a sustainable health workforce [8] and more effective and accessible service delivery [9]. However, change impacting the workforce is often limited to the local level if existing organizational governance structures remain intact and reinforce the status quo [10].

Inconsistent definition and operationalization of the concept of governance has been noted [11]. Broadly speaking it encompasses a whole range of structures and processes through which policies (formal and informal) are enacted to achieve goals, including legislation, regulation and oversight, accountability structures, incentives, and policies to set and maintain strategic direction [12, 13]. In the context of health systems, governance has been characterized as a set of tasks and functions largely established to carry out health ministry goals [14] – essentially driving the direction, type, and accountability of service delivery to improve health system performance [15].

Although there has been increasing interest in governance and health system transformation, as evidenced by a growing literature on the topic, there is still a significant gap in knowledge about how particular ‘tools’ or mechanisms of governance work, in what context, and how they impact health system actors – particularly the health workforce [11]. Pulling together existing, diverse evidence on governance mechanisms and health workforce outcomes will provide decision makers with a better basis for planning future initiatives [11].

The objective of this systematic review was to increase our understanding of the evidence linking health system governance mechanisms to health workforce outcomes. The research questions guiding the systematic review were:

1. How are workforce outcomes accounted for in governance mechanisms in Canada and internationally?
2. What is the impact of governance mechanisms on health workforce outcomes to support health system change?
3. What elements of governance mechanisms are critical to workforce outcomes?
4. How do health system governance mechanisms facilitate workforce changes and contribute to health system change?

## Methods

Early involvement of an advisory committee composed of decision makers and experts in the fields of healthcare policy, governance, healthcare performance, and health workforce was instrumental to ensure the relevance of the review. They helped shape the research questions, refine the literature search, validate the findings, and identify knowledge dissemination opportunities.

The search strategy was developed in collaboration with a university-affiliated research librarian with extensive knowledge of the healthcare databases. Broad search terms related to governance (e.g., governance, hospital administration, leadership, management, missions, models, health care reform) and workforce (based on a list of clinical occupations) were used to capture as many potentially relevant papers as possible (see Additional file 1 for a sample search strategy; see full report for complete strategy [16]). Eligibility criteria were i) publications discussing health systems in Canada, Sweden, the United Kingdom, the Netherlands, New Zealand, Australia, or the United States, ii) published between 2001 and 2012, iii) English or French language, iv) included health workforce (regulated or unregulated healthcare providers) *and* included some form of governance. These countries were chosen because each has features similar to the Canadian system, with the exception of the United States. We included American literature to avoid excluding the vast majority of research on governance in healthcare.

The research librarian executed the search strategy. The following databases were searched for peer-reviewed literature: Medline (OVID), Cochrane CENTRAL Register of Controlled Trials (OVID), Health Technology Assessment HTA (OVID), Cochrane Database of Systematic Reviews (OVID), EMBASE (OVID), PsycINFO (OVID), CINAHL (EBSCO), ABI Inform (ProQuest), Business Source Premiere (EBSCO), ERIC (EBSCO). ProQuest Digital Dissertations, Canadian Research Index (ProQuest), Web of Science Conference Citations, Canadian Health Research Collection (Ebrary) were searched for grey literature (i.e., materials found in sources other than traditional peer-reviewed research, such as conference proceedings and government websites). Both manual and Google site-specific searches of various government and research agency websites were also conducted.

Abstracts of peer-reviewed and grey literature were screened by four raters for inclusion based on abstract rating criteria (see Additional file 2). These were assigned as follows: Yes* (3 points) to abstracts that definitely informed the research questions and that met the search criteria outlined above, Yes (2 points) to abstracts that were likely to inform the research question, Possible (1 point) to abstracts that might possibly inform the research questions, or No (0 points) to abstracts that did not inform the review questions. Inter-rater consistency was established by pre-testing 200 peer-reviewed abstracts and discrepancies were discussed among the four raters until agreement was reached. Following this, we independently screened the entire set of abstracts. Percent agreement among the four raters for the peer-reviewed abstracts was 77% and for grey literature 93%. Raters’ scores were summed and full text articles for abstracts scoring at least five points (of a possible 12) were retrieved for further review. Abstracts scoring four points were discussed among the raters to determine inclusion or exclusion. Two researchers reviewed the full text articles for relevancy using the abstract inclusion criteria. If the two raters disagreed on inclusion, articles were discussed among the four abstract raters until agreement was reached.

Articles retained at the screening stage were then categorized using a classification sheet. The classification sheet itemized the country of study, governance mechanism reported, workforce issues discussed and type of research (i.e., empirical, non-empirical), to simplify later extraction.

These articles were read independently by two researchers and rated for quality. The quality-rating criteria for peer-reviewed empirical articles included items pertaining to the methodological soundness of the study (e.g., clear description of the sample, systematic approach to data collection, valid and reliable measures, appropriate analyses and interpretation). Peer-reviewed non-empirical articles and the grey literature were rated on quality of argument, recency, and originality of the ideas discussed. Peer-reviewed empirical and non-empirical quality ratings also included assessments of the risk of bias in each study, based on whether sources of funding were acknowledged and whether at least one author was unaffiliated with the organization under study (one point each). Quality scores were averaged across raters. Empirical articles achieving a minimum average score of 10 (of a possible 17) and non-empirical and grey articles scoring a minimum of 5 (of a possible 10; see Additional file 3 for quality rating sheets) were retained. We considered scores between 15 and 17 points to be high quality, scores between 12.5 and 14.9 points to be medium quality, and articles scoring in the 10–12.4 point range to be a low quality of evidence. In cases where the two researchers’ ratings differed by more than three points, a third rater also completed the quality rating and all three scores were averaged. Non-empirical and grey papers scoring 8 points or higher were considered to have high quality evidence, papers scoring 7 to 7.9 were medium quality, and scores between 5 and 6.9 were considered low quality evidence.

Bibliographies of retained articles and systematic reviews were screened and potentially relevant empirical articles were retrieved for screening and quality rating to determine inclusion. Following the abstract and article screening stages, the authors thematically sorted the remaining papers into groups of similar topics for analysis. No articles were excluded at this stage; all articles meeting quality and relevancy criteria were all retained for analysis.

The empirical papers included in the review used diverse research methods. Relevant information was extracted into narrative summary tables created for this review [17]. The tables contained fields for author information, country of interest, level and type of governance, workforce details and outcomes, method, results, and additional information. The narrative summary tables were completed by one researcher and validated by a second researcher. Similarly, narrative summaries of pertinent information from the non-empirical and grey literature were summarized by one researcher and validated by a second researcher (see Additional file 4 for sample non-empirical summaries). Once the extraction templates were fully populated a researcher led the writing of a section of narrative synthesis on an agreed thematic heading and the synthesis was then validated by the team.

## Results

**Figure 1:**
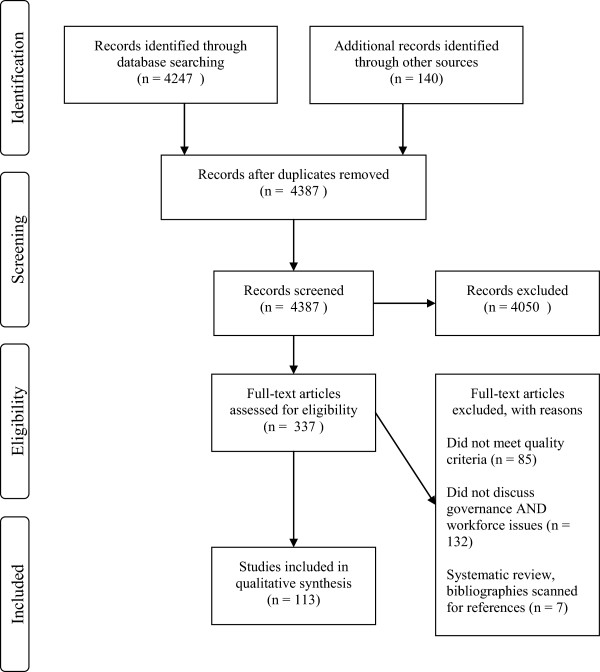
**PRISMA flowchart.**

A PRISMA flowchart detailing the numbers of studies at each stage of the literature search is provided in Figure 1.

Although the team used broad search terms to try and identify a wide range of evidence, our requirement that papers should consider governance mechanisms *and* workforce meant most papers were excluded. In summary, only 113 articles included information on governance mechanisms and workforce and met quality criteria. Forty-nine papers were empirical, 26 were non-empirical, and 38 were grey literature.

### Governance mechanisms identified

Through the review and thematic grouping of papers (as described earlier) six distinct governance mechanisms emerged: shared governance, Magnet accreditation, professional development and education, quality-focused initiatives, reorganization of healthcare delivery, and funding schemes. This article will focus on the first five themes. Results for funding schemes will be presented elsewhere due to the large volume of literature on the topic.

#### Shared governance

Eight empirical articles measured outcomes of shared governance (see Additional file 5). Three reported shared governance was positively related to empowerment [18–20]. Attree [21] noted that nurses in a facility with few opportunities for professional autonomy (i.e., no shared governance) felt disempowered by their lack of influence over practice. Similar results were found for job satisfaction; Ellenbecker et al. [22] found that shared governance was the only retention strategy employed by a sample of home care agencies that increased satisfaction. Attree [21] found dissatisfaction arising from the lack of a true shared governance structure. Frith and Montgomery [20] found improvements in nurses’ relationships with coworkers, physicians, and managers as a result of a shared governance program. Results for retention were less clear; although one study found improvements in retention one year post-implementation [20], another [22] found that shared governance had no effect.

Two empirical studies discussed factors that support the successful implementation of shared governance [20, 23]. Both noted the importance of support for shared governance from management, along with clear communication between staff and administration. This point was repeated in the non-empirical literature [24–28].

Anderson [24] developed a measure of shared governance that could be used to determine whether shared governance was actually in place. The Index of Professional Nursing Governance [24] was administered in one hospital over time, and results suggested that staff nurses and management were in agreement that the hospital did in fact have a shared governance structure, but that it was not developing at the pace they had anticipated.

Winslow et al. [27] described a five-level clinical laddering program developed by nurses in a shared governance program to recognize and reward accomplishments of bedside nurses and motivate them to continue skill development. Although the methodology was not described, they found higher levels of satisfaction among nurses on the advanced levels of the ladder than among nurses on the first two levels. Turnover was substantially lower among nurses on higher levels (less than 1% for levels 3 to 5 vs. 19% at levels 1 and 2). Turnover was also reported to be lower among all nurses involved in the laddering program than among those not in the program. Nurses were satisfied with the laddering process itself and appreciated the opportunity to challenge the level at which they were placed based on their practice. Smith Randolph [29], however, tested whether clinical laddering impacted career satisfaction and desire to stay on the job among several allied health professions and found no effect of laddering on either of these two work attitudes.

The quality of papers in the shared governance literature was mid-grade. One empirical paper scored in the high-quality range, three were of low quality, and four were in the medium range. The non-empirical literature, on the other hand, was primarily low quality: four papers scored in the low range, and just one was medium quality.

### Magnet accreditation

Five empirical articles examined Magnet accreditation (see Additional file 6). Four of the articles compared nursing outcomes in Magnet hospitals to those in facilities without such accreditation. Magnet hospitals have higher percentages of RNs and better Safe Practice survey scores than do non-Magnet hospitals [30]. Although the results were not uniformly supportive of the Magnet model’s superiority for improving nurse outcomes, the slight majority suggest that Magnet status has certain advantages. Two [31, 32] of the three articles examining job satisfaction found higher levels among nurses in Magnet hospitals than among nurses in non-Magnet hospitals. The exception was Hess et al. [33], who found similar satisfaction ratings across Magnet and non-Magnet hospitals. In interviews with senior executives in a hospital applying for Magnet accreditation, Balogh and Cook [34] found evidence of improved staff morale and internal networks. These authors did note, however, that because the interviews were conducted during the accreditation application process, there may be some bias in the results.

Only one article from the grey literature discussed Magnet accreditation. The New Zealand Ministry of Health [35] described an initiative to develop Magnet characteristics in the country’s hospitals in order to reduce staff turnover and burnout and improve recruitment, nurse job satisfaction, and nurse injury rates. Information about the success of the initiative was not available.

The empirical papers that discussed Magnet accreditation were of mixed quality. One paper scored in the high range, two were medium, and two were low quality. The grey article included in this section was in the low range for quality.

### Professional development and education

Seven empirical articles discussed professional development and education programs (see Additional file 7). In general, the training programs resulted in positive outcomes, except in a study by Smith Randolph [29] which found no effect of continuing education on career satisfaction or desire to stay on the job. MacDonald et al. [36] studied a training course designed to enhance collaborative practice. Most learners felt the course increased their confidence in collaborative practice, helped them apply new skills and knowledge in the workplace, and improved collaborative practice. However, there was no change in team members’ attitudes toward teamwork. Garrard et al. [37], while studying a nationwide Hepatitis C training program, found increases in knowledge and confidence about screening, diagnosis, treatment, and patient follow-up. These authors also surveyed participants at one, three, and six months post-training and found that all 28 sites reported at least one major change after one month (e.g., increased communication and collaboration with mental health staff) and that by six months, more than half the sites reported continued improvements in treatment protocols. George et al. [38] examined a training program intended to improve shared leadership in nurses. Positive results were found in pre- and post-program self- and peer-assessments of leadership behaviours in a sample of nurses across five hospitals. Interviews in the months following the training revealed that nurses felt more capable of meeting patient needs and promoting faster recovery, as well as an increased sense of personal growth. They also saw themselves as resources for other staff after the training and noted that better coworker relationships had developed as a result of workflow changes after the training.

The effectiveness of continuing education mandates was the subject of two empirical studies. Results were somewhat conflicting; Prater and Neatherlin [39] surveyed nurses with mandatory continuing education requirements and found they attributed a significant portion of their improvements in various skills to participation in mandatory training. They also had a generally positive view of mandatory continuing education. Smith [40], on the other hand, compared nurses with and without continuing education mandates and found very few meaningful differences in self-rated ability, growth in professional abilities, or hours spent in relevant continuing education courses. Nurses with and without continuing education mandates in this study also made very similar attributions about the sources of their professional growth.

McCabe and Garavan [41] examined the effects of organizational support for staff training and found that nurses’ commitment and motivation were improved by administrative support of training. The non-empirical and grey literature reinforced this point. Narayanasamy and Narayanasamy [42] discussed staff development programs in the UK’s National Health Service (NHS), noting that the organization needs to be supportive, fair, and transparent about staff development and that development plans should be based on accurate appraisals of employee needs. They also argued that development policies should be harmonized with NHS strategies for staff development in order to create a truly supportive organizational culture.

Only one empirical paper discussing professional development scored in the medium range; the remainder were in the low range of acceptability for inclusion in this review. All the non-empirical and grey literature papers were in the low range.

### Quality-focused initiatives

Fifteen empirical articles on quality-focused initiatives (see Additional file 8), which include clinical governance, evidence-based practice, or quality improvement initiatives, were included. Many of these examined providers’ attitudes toward the initiatives. In general, providers were supportive of quality initiatives [43–48], although there was often some apprehension about its implementation [43, 45, 49]. Two articles found that quality initiatives changed workloads; one noted a decrease [48] whereas the other noted a significant increase [47]. Only one study [50] found a large percentage of providers – namely dentists – with negative attitudes about a quality initiative. Dentists in the UK felt they were lacking guidance, that costs and time demands were too high, and that the costs of the initiative would encourage dentists to leave the NHS to practice privately. Interestingly, however, only 30% of the dentists surveyed agreed that care quality would *not* be improved by the program.

Five of the included studies examined the effects of training on attitudes towards or understanding of quality initiatives. Four of these found that training increased acceptance and understanding of quality initiatives [44, 51–53]. Sweeney and Ellis [44] also found enhanced leadership skills and better team relationships after training. Levin et al. [51] and Wallen et al. [52] report that training programs increased providers’ use of evidence-based practice. However, it must also be noted that these training programs were associated with increased workload [44, 45], stress [44], and time pressure [44].

Factors found to facilitate implementation of quality improvement programs are the availability of credible evidence [49, 54], the ease of use of the new practice [54], and, most commonly, leadership support [48, 49, 52, 55].

Facilitators of quality initiatives were also found in the non-empirical and grey literature. Leadership and organizational support were noted as being critical to successful introduction and ongoing use [56–62]. Other methods of engaging staff in quality improvement initiatives were to give them some ownership in the program and hold them accountable for its success [56, 57, 61, 63], ensure that adequate information and resources are provided [57, 59, 64], and, as also suggested by the empirical results, provide staff with adequate training [64–66]. Offering incentives to providers to meet quality standards was also suggested as a means to change provider behaviour [64, 65]. The importance of including a performance review process in any quality improvement initiative was emphasized by several authors [63, 64, 66, 67].

Engaging physicians in quality improvement initiatives can be difficult [60, 67]. Some aspects of medical culture are not conducive to quality improvement, such as physicians’ traditional separation from other care providers [67], and physicians may fear a loss of autonomy, power, and status if they are pushed into interdisciplinary care teams as part of quality improvement projects [67]. These authors also argue that physicians are reluctant to follow clinical guidelines as this might inhibit clinical freedom and devalue clinical judgment. Shortt et al. [65] noted that while Canadian physicians are distrustful of healthcare reform attempts by the government, they generally support quality improvement. The challenge, then, is to reconcile these two viewpoints. Reinertsen et al. [60] created a framework to engage physicians in quality projects: hospitals must link their quality agendas to the physicians’ own quality agenda, recognizing that both parties do want the best quality of care. Physicians must be held responsible for quality, and thus specific roles must be played by physicians and they should be involved from the beginning in planning and implementation. These authors also recommended that hospitals should “standardize what is standardizable, no more” (p. 20), and not create complex care protocols with multiple branches for every possible aspect of care. This was echoed by Mohide and Coker [56], who suggested that evidence-based changes to nursing practice need to be readily understandable, practical, and easy to apply.

Common to the non-empirical literature is a sense that quality-focused initiatives have an impact on providers’ feelings of empowerment to make decisions [58, 59, 68, 69]. Quality improvement is also touted as a means to increase retention [68] and providers’ potential for growth [58] by matching staff’s knowledge and capabilities with their work, thus encouraging better engagement of clinicians and more effective ways of working [68].

Quality of evidence in the empirical papers focusing on care quality initiatives was somewhat low. Eight of the papers were in the low range, four were medium, and three were of high quality. All but two of the non-empirical and grey papers were considered to be low quality, with the others reaching only medium quality.

### Reorganization of healthcare delivery

Ten empirical articles examined various aspects of the structure of healthcare delivery (see Additional file 9). Three of these studies discussed the change from care delivered by individual providers to team-based care delivery, and the results suggest that providers have difficulties adjusting to the change. Belling et al. [70] found that professionals involved in interdisciplinary mental health teams in the UK experienced anxiety about role changes and role overlap resulting from a collaborative care model. Lavoie-Tremblay et al. [71] examined interdisciplinary teams in two psychiatric hospitals in Quebec and found that, although providers believed the interdisciplinary teamwork was rewarding and allowed them more flexibility in their practice, there was also an increase in psychological distress associated with the move to team-based care. Expected improvements in outcomes such as social support from superiors, use of evidence, balance between effort expended and rewards received, and workload did not appear, although providers in one of the hospitals did note an improvement in social support from colleagues. Sicotte et al. [72] examined the factors that contribute to intensity of interdisciplinary collaboration in Quebec’s Community Health Care Centres and found that almost none of the structural or managerial characteristics of the program had any effect. Instead, the most important determinants of collaboration were intragroup process variables such as conflict, belief in benefits of collaboration, and social integration within groups.

O’Dowd et al. [73] examined physicians’ work attitudes as a result of a move to co-operative services to cover work outside of normal hours. Most physicians reported improvements to their quality of life, stress levels, and ability to cope with the demands of work. They were also quite satisfied with the other co-op staff, the shift allocation method, independence, and their own confidence for out-of-hours work. However, half of physicians felt overburdened by co-op responsibilities. Almost two-thirds of respondents would prefer a physician-health board partnership be responsible for organization of care, compared to 23% who would prefer the general practitioner take primary responsibility.

Silvestro and Silvestro [74] examined nurse scheduling practices and their effects on nurses. They identified increased staff stress, work-family conflict, low morale, and poor staff-management relations as potential outcomes of poorly designed schedules. They also found that absenteeism, turnover, and difficulties with recruitment could result from improperly designed scheduling processes.

Braithwaite and Westbrook [75] surveyed staff attitudes toward clinical directorates (organizational arrangements through which specific parts of larger hospitals are managed, e.g., medical, surgical, cardiac services) in an Australian hospital. These authors found large variation and uncertainty in staff attitudes, and concluded that staff were unsure about governance in the hospital and were not clear about the purpose, contribution, or effects of clinical directorates.

The remainder of the articles examined differences across organization types. Donoghue and Castle [76] and Castle and Engberg [77] measured the effects of nursing home features on nurse retention and found opposing results. Donoghue and Castle found that for-profit status lowered turnover for Licensed Practical Nurses (LPNs) but not Registered Nurses (RNs) or Nursing Aides (NAs), while membership in a nursing home chain was associated with higher turnover for RNs and LPNs but not for NAs. However, Castle and Engberg found no relation between chain membership and turnover in any group of nurses, but not-for-profit status was associated with *lower* staff turnover for all nurse types. Castle and Engberg also examined the effect of top management turnover on nursing turnover, and found that NAs and RNs (but not LPNs) were more likely to leave when top management turnover was high.

Aarons et al. [78] examined mental health providers’ use of and beliefs in evidence-based practice (EBP) in private versus public agencies. Workers in private agencies were more supportive of EBP than were workers in public agencies, but this did not predict actual use of EBP. Organizational support for EBP, however, did increase its use. This was true across agency types, although private agencies tended to be more supportive of EBP.

The non-empirical and grey literature contained six articles relevant to reorganization of healthcare delivery. Three of these examined physician organizations in the USA. Smith [79] discussed how Accountable Care Organizations impact the quality and type of care physicians provide. Physicians, according to Smith, are often caught in the middle between cutting costs and avoiding liability because the standard of care set by the government does not take into account the realities of cost cutting. Korda and Eldridge [80] discussed the implications of Accountable Care Organizations for nurses, noting that the ability to participate in interprofessional care and teamwork will be of crucial success. Howard [81] described a physician organization’s difficulties with building a large physician network to sustain a multi-state healthcare system. Difficulties arose immediately, but over time, administration realized that they needed to understand the issues facing physicians (e.g., increased workload needed to maintain results, inflation of practice overhead) and began to place new emphasis on items valued by physicians to build trusting, honest relationships.

Research has shown that incivility and other counterproductive behaviours in the workplace have a significant and negative impact on employees, and so Holloway and Kusy [82] developed a Toxic Organization Change System to reduce and monitor this type of behaviour. The system includes policies, standards, review, and education to address toxicity at the organization, team, and individual levels. Importantly, the authors note that incivility will not stop based on simple education programs or termination of offenders; systematic, multi-level, coordinated strategies are required.

Scott and Lagendyk [83] examined interprofessional relationships in primary care networks in Alberta. They studied five such networks and found that geographical co-location, strong leadership, effective communication strategies, and trust were important to good relationships. These relationships, in turn, were crucial for success in quality improvement initiatives and other practice changes.

Hinings et al. [84] discussed the uncertainty involved in system transformations such as the move to regional health systems in Alberta in 1995. They argued that regionalization had substantial impacts on professional identity for healthcare providers, noting that moves to team-based work and changes to professional boundaries involved changes to what providers did, how they were rewarded, and, consequently, how providers saw themselves.

The quality of evidence on the topic of organization of healthcare delivery was relatively low. The majority of empirical papers scored in the low range, two scored in the medium range, and two were considered high quality. Of the non-empirical and grey literature, two papers were considered medium quality and the remainder was in the low quality range.

## Discussion

The purpose of this systematic review was to examine the evidence relating governance mechanisms in healthcare to health workforce outcomes. We identified six governance mechanisms in the empirical literature: shared governance, Magnet accreditation, professional development initiatives, quality-focused initiatives, reorganization of health services (e.g., moves to team-based care, private vs. public organizations), and funding models. Results on the funding models will be reported elsewhere. Responses to our specific research questions are below.

### How are workforce outcomes accounted for in governance mechanisms in Canada and internationally?

Workforce variables were taken into account to varying degrees in emerging governance mechanisms. Table 1 lists the articles reporting relationships between workforce outcomes and governance mechanisms in the empirical literature. A substantial portion of the literature was devoted to examining various work attitudes (e.g., job satisfaction, engagement) in relation to governance mechanisms. Recruitment and retention were also examined, but perhaps to a surprisingly small extent given the workforce shortages some organizations have forecasted for the coming years [7]. Collaborative practice issues were studied primarily in relation to clinical governance, and absenteeism was examined in only a handful of studies. The same was true for role clarity, learning, workload, and skill or staff mix.

**Table 1:** **Outcomes examined in empirical articles**

***Outcome examined***	Shared governance	Magnet accreditation	Professional development	Quality-focused initiatives	Reorganization of healthcare delivery
**Absenteeism**				Som (2007) [53]	Silvestro (2008) [74]
**Adoption of care protocols**		Jayawardhana (2011) [30]	Garrard (2006) [37]	Gerrish (2008) [49]	Lavoie-Tremblay (2011) [71]
				Levin (2011) [51]	
				Melnyk (2010) [46]	
				Wallen (2010) [52]	
**Collaborative practice**		Balogh (2006) [34]	Garrard (2006) [37]	Fitzgerald (2003) [54]	Sicotte (2002) [72]
			George (2002) [38]	Rosengren (2012) [48]	
			MacDonald (2008) [36]	Som (2007) [53]	
**Learning**			Garrard (2006) [37]	Luxford (2011) [55]	Prater (2001) [39]
			MacDonald (2008) [36]	Fitzgerald (2003) [54]	Smith (2004) [40]
**Professional behaviour**	Latham (2011) [23]	Balogh (2006) [34]	George (2002) [38]	Freeman (2004) [85]	Aarons (2009) [78]
				Paxton (2006) [86]	Prater (2001) [39]
				Rondeau (2007) [69]	Smith (2004) [40]
				Sheaff (2004) [47]	
**Recruitment**	Latham (2011) [23]			Som (2007) [53]	Silvestro (2008) [74]
**Retention**	Ellenbecker (2007) [22]	Brady-Schwartz (2005) [32]		Levin (2011) [51]	Castle (2006) [77]
	Latham (2011) [23]			McCormick (2006) [50]	Donoghue (2009) [76]
				Som (2007) [53]	Silvestro (2008) [74]
				Wallen (2010) [52]	
**Role clarity**			MacDonald (2008) [36]		Belling (2011) [70]
**Skill/staff mix**		Jayawardhana (2011) [30]			McCloskey (2005) [87]
					Silvestro (2008) [74]
**Work attitudes**	Attree (2005) [21]	Balogh (2006) [34]	Garrard (2006) [37]	Dean (2004) [45]	Aarons (2009) [78]
	Barden (2011) [18]	Brady-Schwartz (2005) [32]	George (2002) [38]	Freeman (2004) [85]	Braithwaite (2004) [75]
	Ellenbecker (2007) [22]	Hess (2011) [33]	MacDonald (2008) [36]	Gerrish (2008) [49]	Lavoie-Tremblay (2011) [71]
	Erickson (2003) [19]	Upenieks (2003) [31]	McCabe (2008) [41]	Levin (2011) [51]	O’Dowd (2006) [73]
	Frith (2006) [20]		Smith Randolph (2005) [29]	Luxford (2011) [55]	Prater (2001) [39]
	Kramer (2008) [88]			McCormick (2006) [50]	Silvestro (2008) [74]
	Latham (2011) [23]			Melnyk (2010) [46]	
	Smith Randolph (2005) [29]			Murray (2004) [43]	
				Rondeau (2007) [69]	
				Rosengren (2012) [48]	
				Sheaff (2004) [47]	
				Sweeney (2003) [44]	
				Wallen (2010) [52]	
**Workload**		Jayawardhana (2011) [30]		Rosengren (2012) [48]	McCloskey (2005) [87]

These patterns suggest that researchers are missing opportunities to study aspects of health workforce outcomes (e.g., recruitment, collaborative practice) that could be important for the sustainability of healthcare systems and the quality of patient outcomes. The reverse is also true; decisions about new governance mechanisms do not seem to be influenced by research findings as often as they could or should be.

An important finding of our review is that workforce outcomes are often not explicitly considered in governance mechanism planning efforts. Many of the articles we examined were written by academic researchers studying an initiative after its planning phase, rather than by planners intentionally including the impact to the workforce as a factor in the design of governance mechanisms. The majority of initiatives seemed to be ultimately aimed at improving patient outcomes or reducing financial costs (both worthy goals, of course), not explicitly at improving HHR outcomes. Changes for the workforce are implicit in the planning phase (e.g., implementation of a quality initiative will impact how providers work, but the true goal is to improve patient care) but do not seem to be considered in their own right.

### What is the impact of governance mechanisms on health workforce outcomes to support health system change?

Most governance mechanisms identified during this systematic literature review had at least some of the intended effects on workforce outcomes. Shared governance, Magnet accreditation, and professional development initiatives were most consistently associated with increases to empowerment, confidence, and job satisfaction. Although retention was thought to improve with these initiatives, turnover was not well-studied; shared governance had mixed results and no studies measured the impact of Magnet accreditation or professional development on turnover. However, the significant link between job satisfaction and turnover in Brady-Schwartz’s [32] study of Magnet accreditation does suggest that undertaking the processes necessary to attain Magnet status probably impacts retention as well. Unfortunately, the literature we reviewed does not reveal how these mechanisms impact the measured outcomes.

Quality improvement initiatives also tended to improve staff outcomes in the literature reviewed, although there were often some issues related to increased workload and apprehension about the implementation process. Interestingly, the only study to find strongly negative attitudes about quality improvement [50] examined dentists in the UK. Given that training seemed to increase acceptance of quality initiatives in several of the other studies and that many of the dentists felt they were lacking guidance, it is plausible that these dentists might have benefited from additional education on the process and benefits of evidence-based practice.

Making changes to how healthcare delivery is organized had mixed results; moves to team-based care sometimes resulted in increased stress or issues with role clarity [71], but a move to physician co-operative structures improved quality of life and stress levels among most respondents [73]. In two studies examining the effect of organization type (profit vs. non-profit and chain member vs. independent nursing homes), results were inconsistent. However, the Donoghue and Castle [76] study was based on a much larger sample than was Castle and Engberg’s [77], which may account for the discrepant results.

Overall, the evidence is mixed with regard to how well the various governance mechanisms we reviewed work to create workforce change. More research is needed on each of these topics before we can draw strong conclusions about their effectiveness.

### What elements of governance mechanisms are critical to workforce outcomes?

Each of the governance mechanisms we examined did have some effect on the workforce, and there are some critical elements common to all governance mechanisms that should be considered by health systems planning new initiatives. In sum, the elements that seem most important for successful change are: clear strategy and good leadership that focuses on communication and building trust; engagement of stakeholders from early development through implementation and into ongoing monitoring and refinement of new systems; organizational culture that supports the change and allocates resources to facilitate the process (e.g., funding for training); a reasonable pace for change; and flexibility to take account of local context.

Physician leadership and engagement are also important parts of any healthcare initiative. The value of getting and keeping physicians and other staff members involved in any governance mechanism should not be underestimated. Organizations wishing to begin any project should ensure that all relevant stakeholders are involved in planning and implementation, and should consider what they each value when designing the project.

A key topic that was touched on in many articles was the importance of clear, open communication during all stages of change. Communication from upper management about the organization’s mission and values, along with a clear and reasoned explanation of the need for change were identified as crucial aspects of any kind of governance transformation.

There were a few critical elements unique to certain forms of governance. For shared governance, an important aspect to consider is whether shared governance is implemented in name only or whether providers truly feel in control of their practice. Two articles noted that shared governance might not develop as quickly or as fully as originally intended, and this should be taken into account when examining outcomes.

For quality-focused initiatives, staff seemed to be particularly accepting of and more consistent in implementing this kind of initiative when they received training on how to follow evidence-based guidelines and how to find and interpret research evidence. Training did tend to increase workload, stress, and time pressure, however, so management should consider ways to balance training with usual work requirements.

The literature included under Reorganization of Healthcare Delivery covered a wide range of topics, and thus we cannot conclude with certainty that various changes or types of organization are universally positive or negative. However, instilling trust in the workforce was an important factor in these changes. Organizations should also make sure to understand issues facing the workforce and take these into account when designing new care structures.

It is important to note that although many of the studies alluded to the importance of the above elements, we found no evidence that this had been empirically examined and thus cannot draw firm conclusions about whether they will be useful. That said, in general, elements such as stakeholder engagement, appropriate allocation of resources, strong leadership, clear communication, and training for providers should all be given consideration during the planning, implementation, and evaluation phases of any governance mechanism.

### How do health system governance mechanisms facilitate workforce changes and contribute to health system change?

There were some clear findings in the literature but, overall, the evidence on the impact of governance mechanisms on outcomes in the health workforce is patchy. This is partly explained by methodological weaknesses in the research we reviewed, much of which fails to account for the wide range of factors that may affect intervention implementation and outcomes in health systems. It is also a reflection of a lack of connection to theoretical models for change in the existing research e.g., [89, 90]. There is also a case for more in-depth exploration of the contextual influences on transformational change in complex organizations. Richer, theoretically strong research is important for building the evidence base.

### Strengths and limitations of the review

This systematic literature review had a number of strengths. It had a thorough methodological approach. Each abstract was screened according to preset criteria by four researchers and each full text article was rated and screened for relevancy and quality by at least two readers. Extractions and summaries were written and validated by two separate researchers to ensure all relevant information was included.

Another strength of this review was the integration of guidance from knowledge users and health systems experts to ensure the relevancy and usability of the results. These experts were consulted regularly throughout the conception, search, synthesis, validation, and reporting phases of the project.

Finally, our literature search included not only published empirical literature, but also non-empirical articles and grey literature. This allowed us to examine government and health agency reports and consider expert advice on the topics under review.

The primary limitation of this review was the difficulty inherent in conducting a thorough literature search for governance mechanisms. Given the potential breadth of the topic, it is possible that some important topics or articles were missed despite the assistance provided by an experienced research librarian. We attempted to mitigate this limitation by asking our advisory committee to consider whether any other topics or key papers should be included. We also examined the bibliographies of included articles and retrieved empirical papers within our date range that seemed to address our research questions, and we searched for additional articles written by prominent authors.

The literature search was limited to articles from 2001 and newer, which may have excluded relevant literature. However, this ensured modern governance mechanisms were examined. We also limited our review to research from Canada, Sweden, the United Kingdom, the Netherlands, New Zealand, Australia, and the United States of America, which may also have excluded relevant literature.

Although the majority of literature did agree on key points the quality of papers we reviewed was not high enough to draw firm conclusions about many of the topics under consideration. There is a genuine need for high-quality research in most of the areas we covered. We were careful to eliminate papers with serious methodological flaws, but much of the remaining research did not include control groups, before-and-after designs, or other design elements that would allow us to infer causal linkages between governance, workforce outcomes, and health system change. There is a need for validated measurement tools, larger sample sizes, and the use of comparison groups. In-depth research on how local context impacts policy implementation processes would also help to develop the evidence base. Although research on change in health services organizations does exist [88, 90], the articles we reviewed did not incorporate this research to help explain the success or failure of their initiatives or to increase the odds of success in the planning stages. In addition, a segment of the research we reviewed was conducted by individuals working in the organization under study, which raises the question of conflict of interest. Unbiased, methodologically sound research underpinned by a strong theoretical base is sorely needed to allow users to draw strong conclusions about the effectiveness and suitability of any form of governance. Overall, the quality of evidence hampered our ability to draw strong inferences about the effectiveness of the governance structures and processes we reviewed.

## Conclusion

**Figure 2:**
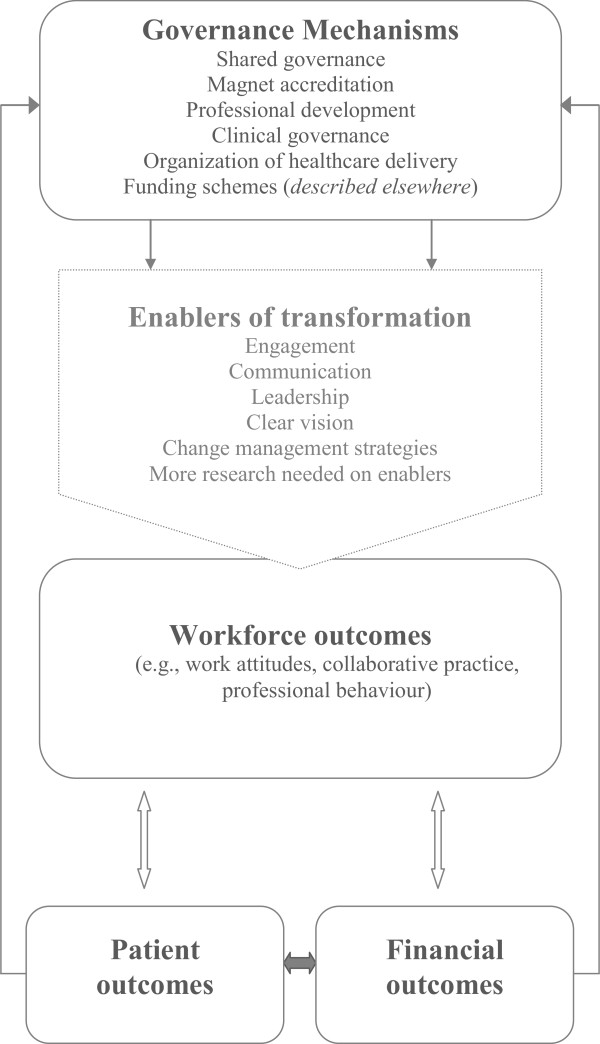
**Workforce outcomes as mediator of relationship between governance mechanisms and patient or financial outcomes.**

The objective of this systematic review was to increase our understanding of the evidence relating health system governance to health workforce outcomes. The lack of high quality, empirical evidence making that link limits our ability to make firm recommendations but we suggest the following for consideration:

- Workforce should be considered as a mediating factor between governance mechanisms and health system outcomes. The literature we reviewed rarely considered both workforce and patient outcomes together. Governance mechanisms that are focused on patient, financial or other system outcomes should include explicit consideration, during the planning, implementation, and evaluation phases, of how the workforce will be affected in order to ensure that the workforce can and will carry out their work in the ways intended (see Figure 2 for an illustration).
- Decision-makers and researchers should work together to develop the evidence base to gain a more complete understanding of the consequences of various types of governance and the mechanisms through which they affect the workforce. Decision-makers and researchers should both advocate for the collection of workforce-related outcomes of governance structures and processes to move research forward in this area.

## Electronic supplementary material

Additional file 1: **Sample search strategy.** (DOCX 15 KB)

Additional file 2: **Abstract rating instructions.** (DOCX 19 KB)

Additional file 3: **Quality rating sheets.** (DOCX 17 KB)

Additional file 4: **Sample non-empirical summaries.** (DOCX 17 KB)

Additional file 5: **Shared governance empirical article extractions.** (DOCX 18 KB)

Additional file 6: **Magnet accreditation empirical article extractions.** (DOCX 16 KB)

Additional file 7: **Professional development empirical article extractions.** (DOCX 18 KB)

Additional file 8: **Quality-focused initiatives empirical article extractions.** (DOCX 25 KB)

Additional file 9: **Organization of healthcare delivery empirical article extractions.** (DOCX 27 KB)
