# Supplementary material for: Exploring the relationship between governance mechanisms in healthcare and health workforce outcomes: a systematic review
Source: BMC Health Serv Res. 2014 Oct 4;14:479. doi: 10.1186/1472-6963-14-479 (PMC4282499; doi:10.1186/1472-6963-14-479)
Supplement: Supplementary file 1 — Additional file 1: Sample search strategy. (DOCX 15 KB) [file 12913_2013_3561_MOESM1_ESM.docx]

Additional File 1. Sample search strategy

**MEDLINE (OVID)**

**Cochrane CENTRAL Register of Controlled Trials (OVID)**

1. Health Manpower/ or models, nursing/ or nursing services/

2. health personnel/ or emergency medical technicians/ or home health aides/ or exp nurses' aides/ or operating room technicians/ or pharmacists' aides/ or physical therapists/ or exp physician assistants/ or exp dental staff/ or exp dentists/ or exp medical staff/ or exp nurses/ or exp nursing staff/ or pharmacists/ or exp physicians/ or exp laboratory personnel/

3. acupuncture/ or chiropractic/ or exp nursing/ or nursing, practical/ or exp nutritional sciences/ or optometry/ or pharmacy/

4. exp Midwifery/

5. exp Naturopathy/

6. exp Occupational Therapy/

7. exp Allied Health Personnel/

8. Allied Health Personnel/

9. exp Podiatry/

10. exp Psychiatric Nursing/

11. exp Nursing Staff/

12. exp Respiratory Therapy Department, Hospital/

13. Social Work/ or patient care team/

14. (acupuncturist* or audiologist* or chiropractor* or clinician* or dental assistant* or dental hygienist* or dentist* or denturist* or dietician* or doctor* or general practitioner* or hearing aid practitioner* or lab technician* or lab technologist* or laboratory technician* or laboratory technologist* or licensed practical nurse* or LPN or LPNs or medical technologist* or medical technician* or diagnostic technologist* or diagnostic technician* or healthcare aid* or healthcare technician* or health care technician* or midwif* or midwives or naturopath* or nurse* or nursing aid* or nursing or occupational therapist* or optician* or optometrist* or paramedic* or pharmacist* or physical therapist* or physician*or podiatrist* or poediatrist* or psychiatric nurse* or psychologist* or registered nurse*or respiratory therapist* or social worker*or speech language pathologist* or surgeon* or therapy assistant* or x-ray technician* or x-ray technologist* or allied health personnel* or provider* or hospital staff).tw.

15. (health workforce or health personnel* or healthcare workforce or hospital personnel or health worker* or healthcare worker*).tw.

16. ((health care or healthcare) adj10 governance).tw.

17. 1 or 2 or 3 or 4 or 5 or 6 or 7 or 8 or 9 or 10 or 11 or 12 or 13 or 14 or 15 or 16

18. exp canada/ or exp great britain/ or exp ireland/ or sweden/ or netherlands/ or new zealand/ or exp australia/ or london/ or exp united states/

19. (canada or canadian* or united kingdom or great britain or england or scotland or ireland or wales or british or UK or uk or sweden or swedish or netherlands or dutch or new zealand* or australian* or australia or united states or usa).tw,in.

20. 18 or 19

21. 17 and 20

22. limit 21 to yr="2001 -Current"

23. limit 22 to animals

24. limit 22 to (animals and humans)

25. 23 not 24

26. 22 not 25

27. governance.tw.

28. *clinical governance/ or *governing board/ or *hospital administration/

29. *models, organizational/ or *decision making, organizational/

30. *"organization and administration"/

31. (govern or governing).tw.

32. (administra* adj5 (power* or function*)).tw.

33. ((administrative or leadership or managerial or management or organisation* or organization*) adj5 (decision making or framework* or missions or model or models or philosoph* or policy or policies or practice* or processes or structure*)).tw.

34. 27 or 28 or 29 or 30 or 31 or 32 or 33

35. Health Care Reform/

36. organizational innovation/

37. ((health or healthcare or hospital* or organization* or organisation* or workforce or work force) adj5 (transform* or change or changing or innovat* or reform* or remodel* or reconstruct* or renewal or restructur* or revolutioni* or transfigur*)).tw.

38. ((quality or performance) adj10 (improv* or innovat*)).tw.

39. 35 or 36 or 37 or 38

40. 34 and 39

41. 26 and 40
