# Supplementary material for: Exploring the relationship between governance mechanisms in healthcare and health workforce outcomes: a systematic review
Source: BMC Health Serv Res. 2014 Oct 4;14:479. doi: 10.1186/1472-6963-14-479 (PMC4282499; doi:10.1186/1472-6963-14-479)
Supplement: Supplementary file 2 — Additional file 2: Abstract rating instructions. (DOCX 19 KB) [file 12913_2013_3561_MOESM2_ESM.docx]

Additional File 2. Abstract rating instructions

Definition of governance from grant proposal:

*There are many definitions for health system governance; some authors define health system governance as encompassing the strategic policy frameworks, mechanisms, effective oversight, coalition building, accountability, information, regulations, and incentives as they relate to health system design. Others refer to health system governance as the actions and means adopted by society to organize itself in the promotion and protection of the health of its population. Ramsay et al. have outlined several key levels and characteristics of health system governance. They differentiate between external levels of governance (e.g., the mandates and strategic planning of regulatory bodies, unions, regional health authorities, accreditation, provincial Ministry’s of health) and local levels of governance which include the strategic plans, committees, quality assurance systems and other management structures and processes at the level of the organization (e.g., hospitals, clinics). These two formal levels of governance are contrasted to informal governance factors such as the relationships between professional cultures, the presence of local champions, and leadership*

1. Read over the questions for the review to re-familiarize yourself with its purpose. As we go through the abstracts, we are trying to flag abstracts that will inform these questions.

- How is workforce transformation accounted for in emerging health system governance models in Canada and internationally?
- How do these emerging governance models facilitate workforce transformation and contribute to health system change?
- What are the elements of governance structures and processes that are critical to workforce transformation?

1. Rate each abstract according to the following scale:

• Y* (definitely informs the review questions; 3 points)

- Y (informs the review questions; 2 points)

• P (might possibly inform the review questions; 1 point)

• N (does not inform the review questions; 0 points).

1. Use the attached relevancy rating criteria to help determine the Y*, Y, P, or N classifications.
2. Input your judgment (either Y*, Y, P or N) on the article rating sheet. Please include any thoughts or comments relating to your rating on the sheet.
3. Respond according to your initial instincts rather than agonizing about your decision for each abstract. You should be re-reading very few. Remember that at least one other person will be rating the same set of abstracts. Even though we will all be “imperfect raters”, the most important papers will tend to rise to the top through multiple ratings.

**Instructions for Rating Abstracts**

Total number of abstracts to be reviewed is 1028 for Medline. Four raters will review a portion of the abstracts. After the ratings are tabulated, all instances of discrepancy (2 or 3 Yes and 1 or 2 No) will be reviewed.

ABSTRACT RATING CRITERIA

***YES** and *YES* Abstracts**

The abstract contains the following elements:

- Based on work in Canada, Sweden, UK (i.e., England, Scotland, Wales, Ireland), Netherlands, New Zealand, Australia, or USA
- Focus is on governance *at higher than unit level*
- Discusses governance, clinical governance, governing board(s), hospital administration, organizational models, organizational decision making, or organizational structure IN COMBINATION WITH health care reform, organizational innovation, quality improvement, or performance improvement
- Inclusion of health workforce/HR considerations
- Be published between 2001 – 2012

**POSSIBLE Abstracts (P)**

The abstract meets one or more of the following conditions:

- The abstract meets most of the Yes criteria but is not clearly relevant
- The abstract does not have sufficient information.
- No abstract

***NO* Abstracts**

- The paper does not consider the health workforce
- The paper only addresses the impact of governance on patient outcomes, quality assurance, service delivery (e.g. access or availability of services), or quality of care.
- The paper addresses unit-level governance (e.g., mentoring programs or medication safety programs implemented on unit, not hospital-wide)
- The research was conducted before 2001.
- The research was conducted in countries other than Canada, Sweden, UK, Netherlands, New Zealand, Australia, or USA
