# Supplementary material for: Exploring the relationship between governance mechanisms in healthcare and health workforce outcomes: a systematic review
Source: BMC Health Serv Res. 2014 Oct 4;14:479. doi: 10.1186/1472-6963-14-479 (PMC4282499; doi:10.1186/1472-6963-14-479)
Supplement: Supplementary file 3 — Additional file 3: Quality rating sheets. (DOCX 17 KB) [file 12913_2013_3561_MOESM3_ESM.docx]

Additional File 3: Quality Rating Sheets

**Governance KS – Empirical Article Quality Rating Sheet**

Reader’s initials:

Article #:

First author surname / year of publication: /

**Section A: Methodological Quality**

Score

0 = not present

1 = present but low quality

2 = present and medium quality

3 = present and high quality

___ 1. **Literature review**

- directly related recent literature is reviewed
- research gap(s) identified

___ 2. **Research questions and design**

- hypotheses, a research purpose statement, and/or a general line of inquiry are outlined.
- study design or research approach is articulated.

___ 3. **Population and sampling**

- the setting is described in detail
- sample type is clear
- participants are described in detail
- approach to sampling is described in detail
- participants’ anonymity and confidentiality were assured and maintained

___ 4. **Data collection and capture**

- key concepts/measures/variables are defined
- systematic approach to data collection is reported
- measures used, if any, have been validated and are reliable
- response or participation rate and/or completeness of information capture is reported.
- methods/interventions are described in sufficient detail as to be replicable

___ 5. **Analysis and reporting of results**

- an approach to analysis and a plan to carry out that analysis is specified; statistical analyses, if any, are appropriate for the study design
- description of results is clear and comprehensive
- adequate sample size was used
- conclusions follow logically from findings
- potential confounding variables are noted

___ / 15

**Section B: Researcher Bias/Conflict of Interest**

Score

0 = No

1 = Yes

___ At least one researcher is not affiliated with facility or organization under study

___ Sources of funding are disclosed

___ /2

Total score: ­­­­____/17

**Non-empirical Article Quality Rating Sheet**

Reader’s initials:

Article #:

First author surname / year of publication: /

Section A: Quality

*Circle appropriate score*

| **Description** | **Decision** | **Score** |
| --- | --- | --- |
| - directly on topic - progressive - evidence of critical thought - strong conceptualization - leading edge - pre-eminent, ground-breaking paper by leading researcher in field - prestigious journal - very recent (2008-2012) | **Critical to include** | **10** |
| - on topic - raises new issues - highlights some interesting ideas - quite good - good journal - quite recent | **Definitely include** | **9**  **8** |
| - relevant and a few interesting ideas - of average interest - not sure of authors credentials - not sure about the journal - mid-date range (2003-2007) | **May reinforce key ideas; perhaps should include** | **7**  **6**  **5** |
| - 1 or 2 interesting ideas, but not innovative - author has strong ties to/is employed by organization under consideration - fairly unknown journal and authors - a bit stale or ideas covered in more recent material - redundant | **Will not be missed** | **4**  **3** |
| - barely relevant - poor writing style - poor logic - local experience - narrow frame of reference - obscure journal - commentator with low-level, non-research related credentials - at old edge of date range (2001-2002) | **Best not to include** | **2**  **1** |

**Grey Article Quality Rating Sheet**

Reader’s initials:

Article #:

First author surname / year of publication: /

Section A: Quality

*Circle appropriate score*

| **Description** | **Decision** | **Score** |
| --- | --- | --- |
| - directly on topic - progressive - evidence of critical thought - strong conceptualization - leading edge - pre-eminent, ground-breaking paper by leading researcher in field - very recent (2008-2012) | **Critical to include** | **10** |
| - on topic - raises new issues - highlights some interesting ideas - quite good - quite recent | **Definitely include** | **9**  **8** |
| - relevant and a few interesting ideas - of average interest - not sure of authors credentials - mid-date range (2003-2007) | **May reinforce key ideas; perhaps should include** | **7**  **6**  **5** |
| - 1 or 2 interesting ideas, but not innovative - a bit stale or ideas covered in more recent material - redundant | **Will not be missed** | **4**  **3** |
| - barely relevant - poor writing style - poor logic - local experience - narrow frame of reference - commentator with low-level, non-research related credentials - at old edge of date range (2001-2002) | **Best not to include** | **2**  **1** |
