# Supplementary material for: Exploring the relationship between governance mechanisms in healthcare and health workforce outcomes: a systematic review
Source: BMC Health Serv Res. 2014 Oct 4;14:479. doi: 10.1186/1472-6963-14-479 (PMC4282499; doi:10.1186/1472-6963-14-479)
Supplement: Supplementary file 4 — Additional file 4: Sample non-empirical summaries. (DOCX 17 KB) [file 12913_2013_3561_MOESM4_ESM.docx]

**Additional File #4**

**Governance KS – Non-empirical Article Summary Sheet**

Reader’s initials: ---

Article #: 2-452

First author surname / year of publication: Weston, 2009

Governance type: Shared governance

Level: Facility

**Summary of major points**

- Managing and facilitating innovation support and reinforce managing and facilitating nurse satisfaction – infrastructure and processes to innovate, adopt best practices, and support nurse satisfaction are mutually supportive
- By definition, engaging employees in innovation involves participative decision making that consequently broadens the diversity of perspectives and contributes to a larger set of alternatives and ideas to be considered. To maximize innovation and nurse satisfaction, degree to which nurses are involved in decision making should be optimized
- Shared governance and other org frameworks and committee structures are effective in creating control over nursing practice when nurses participate, are accountable, and have the authority for addressing and innovating in nursing practice and professional issues. In settings where nurses participate in decision making and are encouraged to innovate, their professional role expands beyond that of caregiver to designer of the care environment, innovator for process improvement, and implementer of practice changes, thereby increasing nurses’ satisfaction
- Innovation is dependent on attracting and retaining talented employees
- Manager leadership behaviors are important both for attracting/retaining employees and for encouraging innovation. Managers need to balance solidarity (necessary cooperation between individuals to focus and accomplish a task) and sociability (affective relationship between individuals typically associated with innovation such as mutual helpfulness, sharing of ideas, valuing on equal terms)
- Success in disseminating innovation requires a systematic, ongoing strategy, including clearly designating the person responsible for innovation dissemination, collaborative opportunities for learning about improvements from others, communication to frontline employees on the innovation to be adopted, and allocation of resources for generating and testing innovative ideas

**Governance KS – Non-empirical Article Summary Sheet**

Reader’s initials: ---

Article #: M500

First author surname / year of publication: Newhouse, 2007

Governance type: Clinical governance

Level: Facility

**Summary of major points**

- Effective nurse leaders are a significant force in the success of evidence-based practice (EBP) because they allocate the human and material resources that provide the context for nurses’ work environment and shape the culture for resource use
- With organizational and management support, nurses’ engagement in EBP often enables their perception of autonomy in practice (Kramer & Schmalenberg, 2004)
- Research indicates that nurses understand the importance of EBP, but lack the required resources within their organizations; perceive that benefits of EBP are high but their knowledge of EBP is low
- EBP can only occur if leaders plan for and provide the org structures and processes, make them transparent and part of normal daily business for clinicians
- If infrastructure to foster EBP is not present (after graduation), the practice environment will not support their continued growth, resulting in lower nurse satisfaction and lower compliance with best nursing practices
- Nurse leaders who understand the value of implementation of EBP to both nurse and patient outcomes will lay the groundwork that serves as a foundation for clinical and administrative practice
- Inviting nurses who are early adopters to help form, strategize, and implement the program will energize change champions
- To encourage adoption, should select staff responsible for creating policy and procedure, develop EBP mentors, recognize staff who are engaging in EBP, outcome reporting of EBP, incorporating EBP into leadership goals
- To embed value for EBP into culture, leaders will need to keep the importance of goals and objectives visible, engage in role modeling, set expectations
- Successful implementation will depend on: the organizational structure, leadership and management, human resource issues, funding, intraorganizational communication and networks, feedback, adaptation, and reinvention.
- Education and training sessions are essential (and ongoing support)
- With leadership support, the structure and processes for EBP are linked to important org priorities, integrated into goals for leaders and nursing committees and job expectations for nurses
- Need strategic plan with goals and timelines (barriers to the adoption and is focused on factors that enhance adoption of innovations)
- The implications for the org and nurses are that the requirement for evidence to drive decisions will result in new competencies, job descriptions, on-going education, and committee responsibilities. An organizational leader who can build infrastructure and enable a culture for EBP will enhance a professional work environment where nurses feel engaged in clinical decisions and base their practice on the best available evidence.
- Effective communication through multiple mechanisms is necessary to help nurses understand goals of EBP program, plan for implementation, and how it will affect them
