# Supplementary material for: Exploring the relationship between governance mechanisms in healthcare and health workforce outcomes: a systematic review
Source: BMC Health Serv Res. 2014 Oct 4;14:479. doi: 10.1186/1472-6963-14-479 (PMC4282499; doi:10.1186/1472-6963-14-479)
Supplement: Supplementary file 5 — Additional file 5: Shared governance empirical article extractions. (DOCX 18 KB) [file 12913_2013_3561_MOESM5_ESM.docx]

Additional File 5. Shared governance empirical article extractions

| **Governance Mechanism** | **Workforce Examined** | **HR Factor(s) Examined** | **Method** | **Results** |
| --- | --- | --- | --- | --- |
| Attree, 2005 UK [21] Quality rating: 12.5/17 | | | | |
| Clinical governance in three hospitals in the UK – intended to devolve control from managers to healthcare professionals (similar to shared governance [SG]) | Registered nurses (RN) | Nurses’ perceptions of their governance, specifically lack of control over factors that affect everyday practice standards;  frustration, dissatisfaction, low morale, demotivation  Also mentioned: turnover, burnout, stress, decreased performance, increased professional negligence | Grounded theory  Semi-structured interviews  Thematic coding  142 RNs from three National Health Services (NHS) hospitals | Outcome category: Work attitudes  Nurses report being individually accountable and responsible for practice standards but without individual control over standards of nursing practice  This perceived lack of autonomy “prevailed across the whole sample and was perceived at all levels of governance above ward level” (p 391)  Nurses described feeling frustration, dissatisfaction, low morale, and demotivation arising from inability to influence factors that affect everyday practice standards  Nurses felt disempowered, lacked “say” in decisions  NHS organizational governance structure characterized by close, central bureaucratic control and positional power, which reduces the opportunity for professional decision making autonomy  No patient outcomes reported |
| Barden, 2011 USA [18] Quality rating: 11/17 | | | | |
| Shared governance (SG) in a New York hospital (details of program not provided) | Nursing | Empowerment  Mentioned importance of empowerment for retention of nursing staff  Discussed downsizing of professional workforce, changes in staff mixes, recruitment, increased workload and responsibilities as result of nursing shortage, job satisfaction, interdisciplinary relationships, autonomy, control over practice | 158 nurses in 13 units from one hospital that had a SG model in place for at least six months to one year  Completed Index of Professional Nursing Governance and the Conditions of Work Effectiveness II Questionnaire (CWEQ-II) | Outcome category: Work attitudes  *r* = .34 (*p* < .0001) between perceptions of SG and empowerment  Study hospital pursuing Magnet accreditation  No patient outcomes reported |
| Ellenbecker, 2007 USA [22] Quality rating: 13.5/17 | | | | |
| Retention strategies used in home care agencies in USA – includes SG/shared decision making  (details of individual programs not provided) | Nursing (home care) | Job satisfaction, intent to stay  Also mentioned: positive work environments supporting nurse autonomy; positive relationships with patients, supervisors, physicians, and peers; workload, job tension, morale, recruitment issues | Survey sent to agency leaders to indicate retention strategies used (n = 123 agencies) and to nurse employees to indicate job satisfaction (Home Healthcare Nurse Job Satisfaction Scale) and intent to stay (n = 2459) | Outcome category: Work attitudes, retention  Shared decision making/SG was the only retention strategy that contributed significantly to job satisfaction scores; no effect of retention strategies on intent to stay  82% of agencies report using shared decision making/SG strategies  No patient outcomes reported |
| Erickson, 2003 USA [19] Quality rating: 12.5/17 | | | | |
| Collaborative governance (decision making  process that places the authority, responsibility,  and accountability for patient care with the  practicing clinician) in large urban teaching hospital in the USA | Nursing | Empowerment  Further discussed: opportunity to develop leadership skills, staff interaction with the larger system, communication across disciplines, dissemination of new knowledge, promotion of new initiatives, decision making across healthcare providers, personal accountability for care, insight into the organization and the roles each professional group played in care delivery, self-growth, respect for unique perspectives, unity | Comparison of empowerment scores over time (baseline, 1-year, 2-year) and across collaborative governance/non-collaborative governance groups  Baseline n = 136  Year 1 n = 292 (134 collaborative governance members, 158 non-collaborative governance)  Year 2 n = 226 (88 collaborative governance, 138 non-collaborative governance)  Instruments used - Conditions of Work Effectiveness Scale (empowerment) along with two extra questions; Job Activity Scale (JAS) and Organizational Relationships scales (ORS) used to measure formal and informal power | Outcome category: Work attitudes  Time analyses:  Empowerment constructs of access to opportunity, information, and resources scores increased each year; access to support was slightly lower at Year 1 but highest at Year 2 (access to resources results not significant [NS]); 2-item empowerment score NS over time  JAS and ORS mean scores increased over time  Groups analyses:  Mean empowerment scores higher at Year 1 and Year 2 for collaborative governance than for non-collaborative governance members  No patient outcomes reported |
| Frith, 2006 USA [20] Quality rating: 12.5/17 | | | | |
| Shared governance (SG) in large medical centre in SE United States (details of structure not provided) | All clinical staff  Registered nurses, licensed practical nurses, care technicians, medical receptionists | Empowerment, autonomy, job authority, accountability, responsibility, retention, turnover, work attitudes  Also mentioned: skepticism of new job authority due to SG; increased knowledge, skill, expertise; increased respect of clinical staff and improved reaction to change by clinical staff; worker cohesiveness, cooperation, and collegiality due in part to increased trust; improved team building, team performance, and teamwork; peer support and commitment; some difficulty motivating staff nurses to participate in SG, difficulty disseminating information to nurses not on councils; mention of increased stress and higher turnover as result of increased time commitment due to SG (though others have found opposite) | Shared Governance Survey sent to clinical staff members (n=687), pre and 1-year post implementation of SG, to assess perception, knowledge and commitment to SG  Some open-ended questions with clinical and managerial staff in four focus groups | Outcome category: Work attitudes  Clear roles, supportive management and effective infrastructure seemed to be most important for the success of SG; Councils needed managers to schedule clinical staff time off for council meeting days and to provide mentorship to the chair  More education and effective communication methods were needed before SG was implemented  SG is time-consuming but effective in meeting identified goals  Improvements found in communication, nursing goals, decision making, educational opportunities, manager/staff partnership, participation in SG, nursing involvement in process improvement, empowerment, co‑worker relationships, MD relationships, retention, excitement about SG, turnover  No patient outcomes reported |
| Kramer, 2008 USA [87] Quality rating: 15.5/17 | | | | |
| Shared governance (SG) in hospitals across the USA (details of individual programs not provided) | Nursing | Control over nursing practice  Empowerment  Also mentioned: cynicism, unwillingness to participate, and reluctance to assume accountability for outcomes when SG is mostly structural and nurses are not given decision making authority; clinical autonomy, collegial nurse-physician relations, job satisfaction, retention; self-determination and self-regulation of profession | Selected 8 highest or second-highest scoring hospitals in country on Essentials of Magnetism instrument to study excellent units in excellent hospitals; selected units based on at least 50% sample and at least five Registered nurses; unit Control over Nursing Practice scores had to be above hospital mean  Conducted interviews, observed participants at meetings, administered CWEQ-II to all staff nurses on participating units  Interviews conducted with 244 staff nurses, 105 nurse managers, 97 physicians from the 101 high-scoring units | Outcome category: Work attitudes  SG was most frequently cited answer to, “What enables you to have control over your practice?”  Comments indicated that interviewees perceived SG structures as sources of formal power  Three hospitals with integrated SG structures (SG structure housed in larger hospital) had higher empowerment scores than the five with silo structures (SG within each unit)  Magnet accredited hospitals  Only brief mention of patient outcomes in background; No patient outcomes reported in findings |
| Latham, 2011 USA [23] Quality rating: 10.5/17 | | | | |
| Shared governance (SG) (Workforce Environment Governance Board) as part of university-hospital mentoring program in two non‑profit acute care facilities in USA | Nursing (Registered nurses [RN]) | Enhanced professionalism, culturally sensitive communication, positive perceptions of workforce environment, support from colleagues, occupational stress levels, unfilled RN vacancies, nurse turnover, shared decision making  Also mentioned: empowerment, autonomy, control over practice (positive work environment involves SG and leads to these outcomes), collaboration, others in relation to mentoring but not SG | 89 mentors and 109 mentees across two hospitals  Baseline and 3-year measurement of occupational stress, cultural competence, perceptions of nursing services and practice environment, existing and desired levels of unit-level decision making by administrators and staff nurses, annual data on RN vacancy and retention rates  Qualitative data obtained through mentor journals and transcriptions of mentor support meetings and governance board discussions | Outcome category: Work attitudes, professional behaviour, recruitment, retention  Most analyses pertain to mentoring program, not SG  For SG: “Mentors and mentees valued having time to meet in workforce environment governance boards and support meetings to collaborate, and this facilitated the sharing of unit procedures and ideas to solve problems. RN staff believed that ongoing interdepartmental sharing by midlevel administrators would help their unit to increase unit-based staff collaboration and support staff activities” (p351).  Governance board also said to improve mentor-administration feedback, thus giving mentors confidence to communicate with and feel supported by the administration.  Reliability of cultural competence measure too low for use; removed from analysis  No patient outcomes reported |
| Smith Randolph, 2005 USA [29] Quality rating: 10/17 | | | | |
| Clinical laddering and continuing education (CE) as extrinsic job satisfaction factors offered by the employer | Occupational therapists (OTs), physical therapists (PTs), speech language pathologists (SLPs) | Career satisfaction, desire to stay on the job  Also mentioned: recruitment, retention | 1500 surveys mailed to practicing OTs, PTs, SLPs , 328 usable questionnaires returned  Surveys measured career satisfaction, desire to stay on the job, and availability and importance of various job factors (e.g. flexible schedule, competitive pay, adequate guidance, clinical laddering, continuing education) | Outcome category: Work attitudes  No significant effect of clinical laddering or CE.  Results revealed that intrinsic factors (those inherent to the job or controlled by the professional) were more important for satisfaction and desire to stay than were extrinsic factors (those controlled by the organization)  No patient outcomes reported |
