# Supplementary material for: Exploring the relationship between governance mechanisms in healthcare and health workforce outcomes: a systematic review
Source: BMC Health Serv Res. 2014 Oct 4;14:479. doi: 10.1186/1472-6963-14-479 (PMC4282499; doi:10.1186/1472-6963-14-479)
Supplement: Supplementary file 6 — Additional file 6: Magnet accreditation empirical article extractions. (DOCX 16 KB) [file 12913_2013_3561_MOESM6_ESM.docx]

Additional File 6. Magnet accreditation empirical article extractions

| **Governance Mechanism** | **Workforce Examined** | **HR Factor(s) Examined** | **Method** | **Results** |
| --- | --- | --- | --- | --- |
| Balogh, 2006 UK [34] Quality rating: 10.5/17 | | | | |
| Magnet accreditation in a National Health Services (NHS) hospital | Nursing, some allied health and medical involvement | Staff morale, internal networks, sharing of good practice, willingness to report poor practice  Also mentioned: improved patient care, excellence in support for professional nursing practice, lower burnout rates, higher job satisfaction in Magnet facilities; address shortages of nursing staff, recruitment and retention | Case study  26 interviews with 10 senior respondents (board members, senior staff) involved in implementing Magnet | Outcome category: Work attitudes, collaborative practice, professional behaviour  Self-report improved morale, new pride in work, improvements in internal networks and sharing of good practice, improved willingness to report poor practice  Authors acknowledge interviews were conducted during accreditation process so may be somewhat biased  No patient outcomes reported |
| Brady-Schwartz, 2005 USA [32] Quality rating: 11.5/17 | | | | |
| Magnet accreditation: Comparison of Magnet and non-Magnet hospitals in the USA (details not provided) | Nursing | Job satisfaction, intent to leave  Also mentioned: recruitment, retention, autonomy, professional development, interdisciplinary relationships, burnout, perception of practice environment, trust in management, empowerment, positive nurse-physician relationships, support for education | 173 Registered nurses (RNs) across three Magnet hospitals, 297 RNs across 3 non-Magnet  McCloskey Mueller Satisfaction Scale – eight facets of job satisfaction  Anticipated Turnover Scale | Outcome category: Work attitudes, retention  Nurses in Magnet hospitals had significantly higher overall job satisfaction than nurses in non-Magnet hospitals  Nurses in Magnet hospitals had significantly higher mean scores on satisfaction with professional opportunities in the work environment, control and responsibility, and extrinsic rewards; no significant differences on praise and recognition, scheduling, balance of family and work life, co‑workers, or interaction opportunities  No analysis of Magnet status 🡪 turnover intention, but results showed relation between job satisfaction and turnover  No patient outcomes reported |
| Hess, 2011 USA [33] Quality rating: 14/17 | | | | |
| Magnet accreditation: Comparison of Magnet vs. non-Magnet vs. in process of pursuing Magnet in the USA | Nursing | Satisfaction with being a nurse, would advise others to become a nurse, injuries sustained on the job, episodes of violence in the workplace, verbal abuse, discrimination, sexual harassment/hostile work environment, decision influence about workplace/patient care, paid education, shared governance (SG) opportunities, quality of professional relationships  Also mentioned: control over nursing practice, opportunities for teaching, role development, professional development, interdisciplinary care and collaboration, workplace safety, mandatory overtime/on-call time, physical demands, influence on decision making | Survey mailed to random sample of 1500 RNs; responses from 175 in Magnet hospitals, 84 in in-process hospitals, 348 in non-Magnet | Outcome category: Work attitudes  Similar ratings of satisfaction with being a nurse across all three  More nurses from in-process or Magnet facilities would advise others to become a nurse  Only slight differences (non-significant [NS]) in reports of violence/abuse/harassment/etc. among each type of hospital  Nurses in Magnet hospitals reported more injuries than nurses in non-Magnet hospitals  Nurses in Magnet or in-process hospitals rated their decision influence on workplace issues higher than non- Magnet, similar (NS) pattern for patient care decisions  More Magnet and in-process hospitals had SG and employer-paid education than non-Magnet  No difference in relationship quality for RNs and new nurses or RNs and physicians across hospital types, but better relationships between nurses and advanced practice nurses in Magnet and in-process hospitals than in non‑Magnet hospitals (Magnet slightly better); relationships between nurses and nursing faculty in in-process hospitals were better than Magnet, and both of these were better than non-Magnet  Remaining comparisons are about hospital characteristics (e.g. size)  No patient outcomes reported |
| Jayawardhana, 2011 USA [30] Quality rating: 16.5/17 | | | | |
| Magnet accreditation: Comparison of Magnet and non-Magnet hospitals across the USA | Nursing | Skill mix (% of RNs), safe practice scores, nurse intensity (nursing hours per patient day)  Also mentioned: autonomy, retention, recruitment, use of evidence-based care, better nursing work environments, lower burnout, higher job satisfaction | Used archival data from Leapfrog Group’s Hospital Annual Survey (2004 – 2006; Safe Practice scores), combined with American Hospital Association Annual Survey and Healthcare Cost Reports Information System  N = 140 (for Safe Practice) or 218 (all other scores) Magnet hospitals and 1320 or 2380 non-Magnet hospitals | Outcome category: Skill mix, care protocols, workload  Magnet hospitals have higher level of nursing intensity (nursing hours per patient day) and higher percentage of RNs than do non-Magnet hospitals  Safe Practice scores are higher in Magnet than in non-Magnet hospitals  Remaining comparisons are about hospital characteristics (e.g. size)  No patient outcomes reported |
| Upenieks, 2003 USA [31] Quality rating: 14/17 | | | | |
| Magnet accreditation: Comparison of Magnet and non-Magnet hospitals in the USA | Nursing | Empowerment, power, job satisfaction (six facets)  Also mentioned: recruitment and retention, control over practice environment, morale | Quantitative: 305 medical/surgical nurses from two Magnet (n = 144) and two non-Magnet (n = 161) hospitals were surveyed; Measured job satisfaction with Nursing Work Index-Revised (autonomy, nurse control over practice, and relations between nurses and physicians subscales in original, three more created for study: administration, self-governance, education opportunities); Empowerment, power (access to information, support, and resources), opportunity (use skill and knowledge, gaining new skills, challenging work opportunities) measured with Conditions of Work Effectiveness II Questionnaire (CWEQ-II)  Qualitative: 16 nurse leaders from same four hospitals (seven from Magnet, nine from non-Magnet) interviewed | Outcome category: Work attitudes  Magnet hospital nurse scores were higher on all subscales than were non-Magnet scores (but similar rankings); Significant differences for all but Opportunity subscale of CWEQ-II  Items “... magnet hospital nurses reported as lacking in their practice environments were adequate support services …, enough time to provide quality patient care, and involvement in the internal governance of the hospital issues.” (p89-90)  Qualitative: organizational culture supportive of nursing influences nurse leader effectiveness; autonomous climate (nurses have control over environment, accountability, authority in decision making) denoted by self-governance systems, decentralization, participatory management, and teamwork (a collaborative approach to patient care through the shared expertise of physicians, nurses, and ancillary personnel) important for supporting nursing practice; access to opportunity (continuing education, clinical ladders, advancement opportunities), adequate staffing, access to resources and information all important for creating positive climate and enhancing nurse leader effectiveness; effective leadership is vital to establishment of cohesive group of nurses and success of hospital  Combined qualitative and quantitative results: Magnet hospitals had greater support from administration than non-Magnet hospitals; Chief Nursing Officers more visible in Magnet hospitals; information more openly provided in Magnet  Differences between Magnet and non-Magnet leaders: Magnet leaders rated as more accessible than non-Magnet leaders; strong commitment to nursing and recognition of nursing practice at Magnet; non-Magnet leaders spoke more of importance of nursing, focused on adequate staffing as crucial element of satisfaction, whereas Magnet leaders stressed educational opportunities  No patient outcomes reported |
