# Supplementary material for: Exploring the relationship between governance mechanisms in healthcare and health workforce outcomes: a systematic review
Source: BMC Health Serv Res. 2014 Oct 4;14:479. doi: 10.1186/1472-6963-14-479 (PMC4282499; doi:10.1186/1472-6963-14-479)
Supplement: Supplementary file 7 — Additional file 7: Professional development empirical article extractions. (DOCX 18 KB) [file 12913_2013_3561_MOESM7_ESM.docx]

Additional File 7. Professional development empirical article extractions

| **Governance Mechanism** | **Workforce Examined** | **HR Factor(s) Examined** | **Method** | **Results** |
| --- | --- | --- | --- | --- |
| Garrard, 2006 USA [37] Quality rating: 12/17 | | | | |
| Nationwide training program by Minneapolis Hepatitis Care Resource Centre – to develop and promote best practices in Hepatitis C (HCV) throughout Veterans Affairs and other healthcare systems  Focus is to increase knowledge and skills of individual participants, to change the interaction or relations within teams, and to provide feedback reviews to help facilitate change within the organization | Mostly nurses and physicians, from both medicine and mental health | Knowledge, confidence, collaboration, treatment protocols  Mention of multispecialty teamwork | 54 participants from 28 sites in training program  Pre/post knowledge and confidence assessments on day 1; follow-up calls at 1, 3, and 6 months with one participant from each site  Eight month training program with needs assessment, 2-day training, 6-month follow-up period | Outcome category: Professional behaviour, work attitudes, collaborative practice  Course was effective in increasing knowledge and confidence about screening, diagnosis, treatment, and follow-up  One month after preceptorship - All 28 sites reported at least one major change related to HCV (e.g. increased communication with mental health staff, increased staff awareness of need for HCV treatment)  End of third month – 19 sites described continued positive improvements, three reported no significant change, three had diminished due to staff being absent or withdrawn  At six month follow-up - 17 sites reported improved treatment protocols  Training program effective in initiating or encouraging collaboration between HCV and mental health staff  End of month 1 – 17 of 23 sites increased contact between provider groups  Month 3 - 16 sites had ongoing or increased collaboration  Month 6 – all sites from months 1 and 3 continued with ongoing meetings, increased communication  Overall learning – if no positive change by end of month 3, it is unlikely there will be changes by month 6  Greatest impediment to change – lack of administrative buy-in and clinician turnover  Some mention of process impact for patients, minor point, not about patient outcomes |
| George, 2002 USA [38] Quality rating: 10/17 | | | | |
| Shared leadership training program –four 8-hour modules over two months intended to increase professional nursing autonomous behaviour; Implemented in hospitals in Wisconsin | Nursing | Staff leadership behaviours, autonomy, staff relations, empowerment, assertiveness, skills, collaboration  Also mentioned: Empowerment, decisiveness, shared vision, motivation, self-efficacy | Study 1: difference in pre- and post-program self-perceptions of leadership in participants and non-participants; 30 participants, 15 non-participants; completed Smola Assessment of Leadership Inventory pre- and post-program (6 months for post)  Study 2: pre- and post- changes in leadership behaviour and professional nursing practice autonomy; 140 nurses from five hospitals; self and peer assessments of Leadership Practices Inventory and self-assessments on Nursing Activity Scale (autonomy) (pre- and 6 months post)  Study 3: perceptions of processes and outcomes associated with development and continued use of leadership behaviours after program; 24 nurses interviewed at 3, 6, 12 months post-completion | Outcome category: Learning, work attitudes, collaboration, care protocols  Study 1: Small increase in leadership perceptions for experimental group (p <.10); no change for non-participants between pre- and post-test; no difference between control and experimental groups at post-test, but authors attribute this to small sample size  Study 2: Statistically significant increases in all five self-reported leadership behaviours and nursing professional practice autonomy between pre- and post-test; peer assessments of leadership behaviours also increased  Study 3: Nurses reported increased ability to meet a variety of patient needs, enhance patient and family trust and rapport with the nurse, improve patient and family satisfaction with care, and promote faster recovery; reported increased personal self-growth (e.g. confident, effective, organized, empowered assertive), less stressed; participated in committees; more effective resources for other staff; better negotiating skills, better relations with others, more accountability for health system; systems improvements decreased workflow issues, and improved team relationships with co-workers, and relationships between nurses and physicians  No patient outcomes reported |
| MacDonald, 2008 Canada [36] Quality rating: 14/17 | | | | |
| Learning/training: ELearning resource designed to enhance collaborative practice (four sections: prepare for collaborative practice, share information, process information, measure collaborative practice [CP]) | Pharmacists, physicians, nurses, nurse practitioners | Reaction to learning experience, acquisition of knowledge and skills about CP, changes in attitudes toward value and use of team approaches to care, learning transfer, increase in interprofessional collaboration, role understanding  Discussed importance of CP for patient care; difficult to implement due to increased workload, differences among staff (e.g. knowledge, skills); need training | 51 learners from three- or four-member teams in long-term care facilities  Three online surveys – Survey 1 (demographics, current knowledge, skills, behaviour, and attitudes toward CP) at first login, Survey 2 (feedback on resource, assessment of whether learning objectives were met, attitudes toward CP) and Survey 3 (CP and impact of learning resource in terms of organizational change and resident well-being) after completion of all learning activities  Also conducted interviews with eight teams at the end of the project (identify strengths and outcomes of the learning resource, provide recommendations for improvement) and interviews with one administrator from each of the eight long-term care homes | Outcome category: Work attitudes, learning, collaborative practice, role clarity  Overall, learners felt resource was beneficial  Interviews and surveys indicated learning objectives had been met, increase in confidence about most CP skills  No change in composite attitudes to teamwork score; one item (team approach permits health professionals to meet the needs of family caregivers as well as residents) changed  82% of learners applied new skills in the workplace as a result of the course; 75% applied new knowledge; 69% initiated new ideas or projects  Team functioning improved, increased understanding of each others’ roles, improved communication, improved CP  Health system change – 49% requested changes be made in their organization to improve care delivery  No patient outcomes reported |
| McCabe, 2008 UK [41] Quality rating: 12/17 | | | | |
| Training and development (no specific initiative, examine organizational support for training and development); Shared governance (not specifically named) – as drivers of commitment in one acute organization and one community organization in the UK | Nursing | Commitment, motivation  Also mentioned: empowerment, training needs, turnover, participation in decision making  Commitment is linked to satisfaction among nursing staff; organizational service orientation is important for performance and service excellence (correlation between commitment and service orientation) | 40 nurses from various wards in two organizations  Semi-structured interviews, grounded theory approach | Outcome category: Work attitudes  Organizational support for staff training – positive message to nursing staff, addressed main drivers and motivational needs of staff, increased commitment  Leadership – motivated and increased commitment of staff, supported staff’s implementation and coping with organizational change, staff expectation that strong senior leadership positively affects line management attitudes which then positively affects staff attitudes  Scope – greater emphasis on specialization, less opportunity for promotion  Shared governance – current system held them accountable without giving them any sense of control (lack of autonomy) or opportunity for involvement in decision making  No patient outcomes reported |
| Prater, 2001 USA [39] Quality rating: 10/17 | | | | |
| Continuing education (CE) mandate by nursing board in Texas | Nursing | Attitudes toward mandatory CE, CE completed, perceived improvement as result of mandatory CE  Also mentioned: increased competency, increased productivity in professional roles, development of new skills and knowledge | Surveyed 123 nurses in Texas  Questionnaire measured attitudes toward mandatory CE, CE completed, demographics, perceived improvements as result of CE | Outcome category: Work attitudes, professional behaviour, learning  Overall attitude toward mandatory CE was positive, but no perceived improvement of psychomotor nursing skills as a result of participation in mandatory CE; positive perceptions related to improvement of cognitive nursing skills, improvement of affective nursing skills, and healthcare of the public; nurses saw increased general knowledge base as most beneficial outcomes of mandatory CE, followed by awareness of professional issues; cost is biggest perceived problem with mandatory CE  Significant positive relationships were found between nurses' attitude toward CE and their perceived improvement in:  Healthcare of the public (r = .52, p < .001)  Affective nursing skills (r = .57, p < .001)  Psychomotor nursing skills (r = .65, p < .001)  Cognitive nursing skills (r = .52, p < .001)  General knowledge (r = .38, p *<* .001)  No patient outcomes reported |
| Smith, 2004 USA [40] Quality rating: 10/17 | | | | |
| Nursing board mandates for continuing education (CE) in the USA (vs. nursing boards without mandates) | Nursing | Development of professional competence, self-rated ability, hours of CE completed | Questionnaire developed for project included questions about 10 professional abilities and questions about issues potentially influencing growth of professional abilities  1025 completed questionnaires (478 from Licensed practical nurses [LPN]/Vocational nurses [VN] and 547 from Registered nurses [RN]) from 35 nursing boards  Comparison of nurses with and without mandated CE | Outcome category: Learning, professional behaviour  Compared self-ratings of ability (retrospective rating of when first began as a nurse and a current rating) for nurses with and without mandated CE; only significant finding for 10 abilities was LPN/VN respondents’ current ability for assessing client or service outcomes (mandated were higher)  Subtracted beginning ability from current ability to measure growth of professional abilities; no statistically significant or practically relevant differences in the amount of growth experienced by either RNs or LPN/VNs with and without CE mandates  Nurses were asked to rate factors contributing to current abilities; no differences found between mandated and non-mandated (most points for work experience, followed by basic professional education)  Slightly more CE hours for mandated vs. non-mandated, but not statistically significant; mandated nurses did complete significantly more hours of CE unrelated to their current work  Employment conditions and other issues influencing growth in abilities: access to CE through their employers was especially problematic for LPNs/VNs in long-term care (48%); 43% RNs and 45% LPN/VN sometimes or frequently NOT allowed time off for CE  No patient outcomes reported |
| Smith Randolph, 2005 USA [29] Quality rating: 10/17 | | | | |
| Clinical laddering and continuing education (CE) as extrinsic job satisfaction factors offered by the employer | Occupational therapists (OTs), physical therapists (PTs), speech language pathologists (SLPs) | Career satisfaction, desire to stay on the job  Also mentioned: recruitment, retention | 1500 surveys mailed to practicing OTs, PTs, SLPs , 328 usable questionnaires returned  Surveys measured career satisfaction, desire to stay on the job, and availability and importance of various job factors (e.g. flexible schedule, competitive pay, adequate guidance, clinical laddering, continuing education) | Outcome category: Work attitudes  No significant effect of clinical laddering or CE.  Results revealed that intrinsic factors (those inherent to the job or controlled by the professional) were more important for satisfaction and desire to stay than were extrinsic factors (those controlled by the organization)  No patient outcomes reported |
