# Supplementary material for: Exploring the relationship between governance mechanisms in healthcare and health workforce outcomes: a systematic review
Source: BMC Health Serv Res. 2014 Oct 4;14:479. doi: 10.1186/1472-6963-14-479 (PMC4282499; doi:10.1186/1472-6963-14-479)
Supplement: Supplementary file 8 — Additional file 8: Quality-focused initiatives empirical article extractions. (DOCX 25 KB) [file 12913_2013_3561_MOESM8_ESM.docx]

Additional File 8. Quality-focused initiatives empirical article extractions

| **Governance Mechanism** | **Workforce Examined** | **HR Factor(s) Examined** | **Method** | **Results** |
| --- | --- | --- | --- | --- |
| Dean, 2004 UK [45] Quality rating: 11/17 | | | | |
| Quality improvement (QI) as part of National Health Services (NHS) clinical governance (CG) initiative (details on programs in individual practices not provided) | General practitioners (GPs), nurses, allied health, administration or reception | Attitudes toward QI  Lack of time, knowledge, skills, benefit from CG all noted as barriers to implementation; deficiencies in teamwork | 192 members of primary healthcare teams from 17 general practices (11 city, 6 rural or market town; 6 teaching or training practices, 6 solo or dual partner practices; 3 were in underprivileged areas)  Sent questionnaires with open-ended items about confidence in QI, beliefs about whether it would be beneficial, any personal anxieties about QI | Outcome category: Work attitudes  Most were confident about practice’s ability to take part in and benefit from quality initiatives  Believed that good team processes (communication, cooperation, enthusiasm, conflict resolution) and selecting appropriate or relevant initiatives to motivate the team were necessary for successful QI; poor team functioning reduced confidence  Team members wanted to be involved in initiatives but had anxieties about whether this would happen (want to be consulted) because of issues with the systems, funding and time  Concerns about QI increasing workload  Article includes frequency and percentages by occupational group of positive, negative, conditional and unknown statements; as well as emergent categories’ statements  No patient outcomes reported |
| Fitzgerald, 2003 UK [54] Quality rating: 11.5/17 | | | | |
| Evidence-based decision making in four health authority areas in the UK (no specific initiative) | GPs, nursing, physiotherapists, non-medical managers, clinical academic, medical manager, Chief Executive Officer (CEO), GP / commissioner, director of public health | Collaborative practice, clinical practice | Comparative, longitudinal case studies (total of 113 interviews with multiple types of staff across four health authority areas – see list in “workforce examined”)  Macro and micro-stage to data collection; macro-phase interviews across four health authorities (chief executive, public health director and primary care lead, GPs); micro-phase interviews (GPs, nursing, physiotherapist, non-medical manager) | Outcome category: Collaborative practice, professional behaviour  Doctors wished to establish the credibility of evidence from the source; nurses demonstrated less willingness to engage directly and tended to receive information from doctors  Professionals weighted factors for spread: robust scientific evidence to support innovation, innovation is applicable, neutral cost implications, new intervention or treatment is not so complicated, intervention raises patient satisfaction  Partnership-based organizations operate in a consensual, non-hierarchical way at the top, while within the practice there are distinct hierarchies between professions; networks need collaborative effort based on consensus; issues relating to the organization of services, control and CG need to be handled differently in primary care  No patient outcomes reported |
| Freeman, 2004 UK [88] Quality rating: 13.5/17 | | | | |
| Clinical governance (CG) initiative by NHS (specific details for each trust not provided) | Board and managers of acute, ambulance and mental health/learning disabilities Trusts | Collaboration and leadership, improving staff performance | Survey of CG in NHS trusts  1916 participants selected across 100 Trusts (1177 returned surveys)  National cross sectional study questionnaire – assesses organizational performance on achievement on organizational competencies related to CG and perceived importance of the competencies | Outcome category: Work attitudes, professional behaviour  Leadership and collaboration were perceived as important (fourth on the list) (mean 8.0, 95% CI 7.9-8.1); improving performance was third (mean 8.1)  Lower perceived achievement in leadership and collaboration (mean 5.6, 95% CI 5.5-5.8)  Biggest differences between perceived importance and perceived achievement were for leadership and collaboration (mean 2.4, 95% CI 2.3-2.5) and improving quality (mean 2.3, 95% CI 2.1-2.4), followed by performance improvement (mean 1.9)  More progress in areas concerned with quality assurance than quality improvement  No patient outcomes reported |
| Gerrish, 2008 UK [49] Quality rating: 11.5/17 | | | | |
| Evidence-based practice (EBP) – various initiatives relating to reduction of pressure damage in two hospitals in England | Nursing (senior nurses, junior nurses) | Research utilization (i.e. use of EBP), knowledge and skills in implementing EBP | Data collected using Developed Evidence-based Practice Questionnaire which is comprised of:  Section 1 – knowledge used by nurses in their practice (adapted from Estabrooks scale, 1998)  Sections 2-4 – barriers to achieving EBP  Section 5 – self-rating on skills of finding and reviewing evidence and using evidence to effect change  All registered nurses (RNs) in two hospitals (excluding those already participating in a study regarding EBP, two clinical directorates in one hospital)  Questionnaires returned:  Hospital 1 n = 330  Hospital 2 n = 274  Combined useable sample N = 598 | Outcome category: Care protocols, work attitudes  Utilization: Nurses tend to draw from experiential knowledge acquired through interactions with patients and colleagues to a much greater extent than formal knowledge from textbooks and journals; knowledge was also gained from doctors, in-service training, and policy and procedure manuals  Barriers: Greatest barriers to research utilization were related to time and availability of information; skill in judging quality of information and identifying implications for practice also of concern; nurses were generally confident about where to locate information  Barriers to changing practice: time and resources; confidence in ability to effect change, lack of authority, non-receptive culture also barriers; also perceived lack of support from managers, colleagues, medical staff for change to practice  Skills: Nurses were more confident in finding organizational information and reviewing organizational information than finding and reviewing research evidence; using organizational information or research information to change practice were the areas of least confidence  Differences between senior and junior nurses: Senior nurses consult formal sources of knowledge (research journals, audit reports, internet) and organizational information, and were confident they could implement change based on EBP;  junior nurses use their education as source of EBP knowledge, and were challenged finding organizational information, implementing change based on EBP  No patient outcomes reported |
| Levin, 2011 USA [51] Quality rating: 16/17 | | | | |
| Evidence-based practice (Advancing Research and Clinical practice through close Collaboration [ARCC] model; involves system wide implementation and EBP mentor to assist others) | Nursing (health home care) | Beliefs about EBP, implementation of EBP, group cohesion, job satisfaction, productivity, turnover, learning | Two group randomized controlled trial with repeated measures (22 nurses in experimental group, 24 in control); nurses in experimental group were given EBP training, toolkits, environmental prompts, EBP mentor  Data collected at 4 time points (Time 1=baseline, Time 2=4 weeks, Time 3=16 weeks, Time 4=9 months after completion)  Turnover rates compared to year before for each group  Learning questionnaire given to both groups to assess knowledge | Outcome category: Care protocols, work attitudes, retention  Statistically significant improvement in ARCC nurses’ EBP beliefs at Times 3 and 4, compared with control group (EBP group increased from Time 1 to 3, slight decrease at Time 4)  Nurses in EBP group demonstrated greater implementation of EBP at Times 3 and 4 than did controls, and EBP increased in EBP group from Time 1 to Time 3  No group effects on group cohesion, but EBP group was more cohesive at Time 3 than Time 1  No effects of group or time on job satisfaction  No effects of group or time on productivity  EBP group turnover (vs. previous year) was reduced by almost 50%; no change for control group  No significant difference between EBP and control nurses on EBP learning questionnaire, but EBP nurses answered more questions correctly in physical assessment portion of exam (both were given this training)  see also Melynk 2010 and Wallen 2010 re ARCC model  No patient outcomes reported |
| Luxford, 2011 USA [55] Quality rating: 13/17 | | | | |
| Strategies to support change to patient-centred care (includes quality improvement; no specific initiative or details provided) in healthcare organizations across the USA | Interviews conducted with senior leaders (e.g. CEO, Chief Medical Officer, quality director), but discuss general staff issues | Employee satisfaction, building staff capacity, accountability  Supportive work environment for all employees, importance of clear communication of strategic vision | Eight healthcare organizations (three acute inpatient hospitals, three medical groups or ambulatory care, two health management organizations) selected for study due to either having widely recognized reputation for improving patient care experience or were high performers in patient care experience data  Five key informants from each site were interviewed (semi-structured) | Outcome category: Work attitudes, learning  Interviewees from 7/8 sites reported that strong committed senior leadership (CEO, governance support), communication of strategic vision (5/8 sites), a focus on improving satisfaction of employees is a facilitator for building patient-centred care; 7/8 identified building capacity of staff to support patient focus (e.g. training on values, communication, customer service)  6/8 incorporate patient feedback into performance reviews to enhance accountability (and pay incentives)  7/8 felt they need to change mindset of employees from provider focus to patient focus  Other learnings – change takes longer than anticipated (5/8), leaders are influential in successful change of culture and employee support, insufficient resources is a barrier  Suggestions – CEOs in study had longer than average tenure which may have supported a strategic long-term approach to QI  Loose discussion of transition to patient centred care (including outcomes) but no reporting of measured patient outcomes |
| McCormick, 2006 UK [50] Quality rating: 11.5/17 | | | | |
| Clinical Governance (CG)  “a framework through which NHS organizations are accountable for continuously improving the quality of their services and safeguarding high standards of care by creating an environment in which excellence in clinical care will flourish” (p214) | Dentists | Attitudes towards CG  Professional performance, recognition and promotion of good practice, identifying and remedying poor practice, understanding of CG, frustration and cynicism about CG, retention and intent to leave | Questionnaire to assess attitudes and opinions towards CG  Questionnaire sent to 208 dental practices | Outcome category: Work attitudes, retention  73% agreed/strongly agreed there was not enough guidance on implementing CG  72% agreed/strongly agreed it takes too much time to implement CG  31% agreed/strongly agreed that quality in practice will not be improved with CG  52% agreed/strongly agreed that cost is a major negative factor in implementing CG  60% agreed/strongly agreed the costs of CG will make more dentists leave NHS practice  No patient outcomes reported |
| Melnyk, 2010 USA [46] Quality rating: 15/17 | | | | |
| Evidence-based practice (EBP; ARCC model) | Nursing, allied health | Attitudes toward EBP (value of EBP, ability to implement it)  Implementation of EBP  Group cohesion  Job satisfaction  Also mentioned: professional autonomy, collaboration, turnover, morale, empowerment | 58 nurses and other health professionals who had been selected to participate in EBP mentorship program as part of implementing ARCC model; measures administered at start of project  Instruments used - Organizational Culture and Readiness for System wide Integration of Evidence-based Practice, Evidence-based Practice Beliefs, Evidence-based Practice Implementation, Group Cohesion, Price and Mueller Job Satisfaction  Questionnaires were given BEFORE implementation | Outcome category: Work attitudes, care protocols  Fairly strong beliefs about EBP and ability to implement it, although level of implementation was relatively low  Participants with stronger beliefs about EBP implemented it to a greater extent, reported higher group cohesion and job satisfaction, and perceived organizational culture as more positive and ready for EBP  see also Levin 2011 and Wallen 2010 re ARCC model  No patient outcomes reported |
| Murray, 2004 UK [43] Quality rating: 14/17 | | | | |
| Clinical governance (CG) – NHS initiative to place quality at the heart of the organization and emphasize need for accountability (specifics of each program not provided) | Nursing, healthcare assistants, support workers, speech language pathologists, physiotherapists, occupational therapists, pediatricians, clinical psychologists, psychiatrists, social workers, music and art therapists, managers and heads of departments, administrative staff | Knowledge, attitudes and implementation of CG  Also mentioned: professional development (as a pillar of CG), need for CG to be accepted by staff for success, lack of time and support, and cultural resistance to change (as barriers to CG) | Staff CG survey to determine staff perceptions about CG and its implementation  539 participants across three NHS trusts  Cross-sectional design, survey research | Outcome category: Work attitudes  Majority viewed CG as useful, clear, welcome, but also complex and tiresome  Good knowledge about CG  Many staff were not aware of who their line manager was and guidelines for confidentiality; this prompted some to find this information  Questionnaire can be used as an audit tool within Trusts and as a research tool to highlight ways in which CG can be promoted  Mean self-rating of implementation was 3.62(/5) but no details provided on what was included in this scale  Knowledge self-rating mean was 3.43, attitude self-rating was 4.02  No patient outcomes reported |
| Paxton, 2006 USA [89] Quality rating: 10.5/17 | | | | |
| Audit and feedback process designed to increase productivity by providing clear clinical performance information in an academic hospital’s department of surgery | Physicians | Productivity (measured in relative value units [RVUs])  Also mentioned: autonomy, responsibility, workload | 69 physicians with sufficient RVU data were included in the sample. Comparison of approx. 18 months pre- and post-implementation of feedback sheets  Physicians were sent survey at the end of experimental time period to measure their usage and perceptions of the monthly performance data (n = 40) | Outcome category: Professional behaviour  Increase in RVUs of 6% after implementation of feedback sheets (after removal of outliers, n=68)  89% of physicians believed the feedback sheets were useful, 92% viewed and used the reports  No patient outcomes reported |
| Rosengren, 2012 Sweden [48] Quality rating: 10.5/17 | | | | |
| Quality registry (Senior Alert [SA]) developed due to need for systematic approach within malnutrition, pressure ulcers, falls) in two hospitals in Sweden | Nursing | Change of mindset, teamwork, work pressure  Also mentioned: importance of managing change by minimizing anxiety for staff | Eight interviews with nurses (one assistant nurse, seven RNs) from two hospitals (one ward per hospital)  Questions based on experiences with implementation process and QI work with the registry | Outcome category: Work attitudes, collaborative practice, workload  Change of mindset from traditional care to QI work through a preventive approach was described  Committed leadership support for change process important (e.g. daily reinforcement of SA, promoted cross boundary collaboration)  Teamwork with different professionals was described as significant and positive in creating synergy  Nurses reported that registry could reduce work pressure as a result of more effective and preventive methods  Challenges to QI – patient needs (e.g. different care providers required for optimal care)  Brief reference to patient outcomes in introduction and discussion; no formal measurement of outcomes |
| Sheaff, 2004 UK [47] Quality rating: 11/17 | | | | |
| Soft governance of primary care groups/trusts over GPs (i.e. use of impersonal management tools and indicators to manage and measure performance, reward accordingly); clinical governance (CG) | Physicians | Attitudes to CG, workload, changes to clinical practice  Also mentioned: threats to professional autonomy | Chose two “excellent” sites, two having difficulty providing mental health services, two that prioritized mental health (MH) but were neither excellent nor deficient, and six “middle of the road” sites  49 semi-structured interviews with key informants (e.g. chief executive, CG lead, MH lead)  Document analysis  437 questionnaires from GPs about awareness of CG activity, attitudes toward it, methods used, and resulting practice changes | Outcome category: Work attitudes, professional behaviour  58% had positive attitude toward CG, 13% negative, 29% neutral  88% said workload had increased, 12% said unchanged, 0.2% said decreased  93% of GPs said their practice had changed because of CG; 48% said it made no difference to their quality of care  No patient outcomes reported |
| Som, 2007 UK [53] Quality rating: 12.5/17 | | | | |
| Clinical governance (CG) (human resource [HR] management [HRM] issues associated with implementation of CG) in a NHS hospital trust in the UK | All staff in NHS trust | Training, recruitment, retention, performance appraisal, absenteeism, multidisciplinary teamwork  Also mentioned: commitment, motivation, and enthusiasm (as drivers of CG in NHS), team-oriented learning activities | Interviews with 33 key informants (doctors, nurses, HR managers, general managers) and document analysis at one NHS hospital | Outcome category: Learning, recruitment, retention, absenteeism, collaborative practice  Participants appreciated the crucial role of HRM in the implementation of CG by putting right people in right positions, providing opportunities for skill upgrades, preparing staff for advanced roles, and ensuring adequate staffing levels in each department  Staff members need to be trained in how to practice evidence-based medicine (EBM), how to monitor own activities and prove they are evidence-based  Need skills in critical research appraisal  Issues with performance appraisal process and implementation – needed for EBM, but hard to implement such a sensitive issue  Issues with recruitment and retention due to funding issues – shortage of staff is the biggest problem in most units studied  Sickness absence further aggravates staffing problems; Trust has comprehensive policy, but it needs improvement; this staff shortage makes others tired, influencing long-term retention  Need more team-oriented learning opportunities to enhance multidisciplinary teamwork  Mention that few articles pay attention to HR implications of CG  No patient outcomes reported |
| Sweeney, 2003 UK [44] Quality rating 11/17 | | | | |
| Clinical Governance Development Programme (CGDP) “aims to inspire and enable frontline teams to demonstrate understanding and application of CG through a patient-centred programme of facilitated change” (p262) | Multiple (e.g. nurses, physicians, allied health) | Attitudes toward CG, workload, empowerment, motivation  Desired HR outcomes of CGDP: enhanced leadership skills, effective team working, embrace CG to improve patient experience | Telephone interview with 500 delegates who had participated in the CGDP; assess impact the program had on participants, their working practices and work relationships | Outcome category: Work attitudes  76% agreed their leadership skills have been enhanced  62% believed they now have better relationships with colleagues  79% agreed their understanding of QI issues increased  Negative impacts reported: increased workload, pressure of time, levels of stress  Main benefit of meeting and sharing with other health professionals, acquiring new skills  Increased feeling of empowerment, heightened motivation in the change process  No patient outcomes reported |
| Wallen, 2010 USA [52] Quality rating: 15.5 | | | | |
| Structured mentorship program to implement EBP (ARCC model) in Maryland hospital | Nursing | EBP beliefs (value and ability to implement), EBP implementation, group cohesion, job satisfaction, intention to leave, intention to stay in nursing  Also mentioned: improvements in care quality and practitioner skills | 159 participants at baseline (94 in EBP implementation group, 65 in non-workshop group) and 99 participants at post-intervention (58 in EBP, 41 in non)  Also ran focus groups (four clinical nurse specialists, nine nurse managers, five members of Shared Governance Clinical Practice Committee)  For EBP group: 2-day intensive workshop to improve EBP knowledge and skills, ongoing mentoring and skill-building activities, online tutorials  Instruments used – see Melynk 2010 + Intent to Leave scale, Nurses’ Retention Index | Outcome category: Work attitudes, care protocols, retention  Focus group participants believed nurses might be resistant to EBP unless it was applicable to their practice; need leadership support and dedication of resources  Survey results: Those who attended EBP workshops had larger increase in EBP Belief scores than those who did not attend, more change in implementation (+), job satisfaction (+), group cohesion (+), and intent to leave (-); no difference for intent to stay in nursing  see also Levin 2011 and Melynk 2010 re ARCC model  Reference to importance of patient outcomes in background, but no reporting of outcomes in findings or discussion |
