# Supplementary material for: Exploring the relationship between governance mechanisms in healthcare and health workforce outcomes: a systematic review
Source: BMC Health Serv Res. 2014 Oct 4;14:479. doi: 10.1186/1472-6963-14-479 (PMC4282499; doi:10.1186/1472-6963-14-479)
Supplement: Supplementary file 9 — Additional file 9: Organization of healthcare delivery empirical article extractions. (DOCX 27 KB) [file 12913_2013_3561_MOESM9_ESM.docx]

Additional File 9. Reorganization of healthcare delivery empirical article extractions

| **Governance Mechanism** | **Workforce Examined** | **HR Factor(s) Examined** | **Method** | **Results** |
| --- | --- | --- | --- | --- |
| Aarons, 2009 USA [78] Quality rating: 16/17 | | | | |
| Private vs. public status of mental health service provider agencies in the USA | Mental health service providers (e.g. social work, psychology, counselling, marriage and family therapy) | Attitudes toward evidence-based practice (EBP)  Use of EBP  Suggested private vs. public organizations may have different norms/expectations around and supports for use of EBP, and that this may have effects on individual beliefs; in turn, beliefs should affect behaviour  Discussed perceived organizational support (fairness, supervisor support, organizational rewards and job conditions) – relates to work outcomes like job satisfaction, improved performance, and greater job involvement | Self-reported measures of EBP attitudes (global attitude toward adoption of EBP) and use of EBP (checklist of 31 EBPs)  170 respondents (41 public, 129 private) | Outcome category: Work attitudes, professional behaviour  Working in private agency was associated with more positive attitudes to EBP; no significant relation between attitudes and use of EBP; agency type predicted attitudes to EBP and organizational support for EBP, but the only mediator of Agency type 🡪 EBP use was organizational support for EBP (i.e. Agency type predicts organizational support for EBP, which in turn predicts EBP use)  Private organizations were more supportive of EBP than were public organizations  No patient outcomes reported |
| Belling, 2011 UK [70] Quality rating: 10.5/17 | | | | |
| Community Mental Health Teams in National Health Service (NHS) mental health trusts in greater London – goal is to provide continuity of care by using mix of professionals working collaboratively from one set of notes | Psychiatrists, psychologists, social workers, nurses, occupational therapists (OT), general practitioners (GP), volunteers | Role and identity  Mentioned carer distress and confusion arising from service discontinuities  For continuity of care, need consistent information given to users and carers, effective coordination of services, development of flexible care plans linked to effective monitoring, deployment of professional staff to remove disjointed episodes of service delivery, designation and accountability of one or more professional staff to foster therapeutic relationships and exert positive impact on care outcomes, and development of systems and processes to provide care adequate to meet needs over time  Mentioned that in early stages of policy implementation, there were concerns over communication, coordination and decision making difficulties, concerns over loss of professional identity, limited resources, lack of time, bureaucracy, and leadership | Semi-structured interviews (n=113)  (majority of respondents in each trust were social workers or nurses) | Outcome category: Role clarity  Some members expressed anxiety at perceived erosion of professional roles and identities due to generic and cross-boundary working  No patient outcomes reported |
| Braithwaite, 2004 Australia [75] Quality rating: 12.5/17 | | | | |
| Clinical Directorates (CDs)– intermediate organizational arrangements through which defined parts of larger hospitals or health services are managed (i.e. grouped services either derived from pre-existing organization of medicine [e.g. medicine, surgery] or how services are delivered to patients [e.g. cardiac services, cancer services]) in large publicly funded hospital three years after conversion to CD | Physicians, nurses, allied health, administrators (all in managerial positions for CDs or units) | Attitudes toward clinical directorates: clinician issues, working relationships, coordination and management issues, decentralization, organizational performance and benefits  Also mentioned: improved efficiency, combining managerial and clinical expertise in useful ways, organizational relationships, group behaviour, collaboration, trust | 107 hospital staff completed survey developed by authors (based on literature and focus groups with 64 clinical unit managerial staff, pilot tested with 40 clinician managers, validated by expert panel of three academics in health sciences)  Survey covers: clinician issues, working relationships in the hospital, coordination and management issues, decentralization, organizational performance and benefits  Management roles: 49 managerial role, five non-CD hospital executives, nine senior CD managers, one business manager, 34 ward unit or department managers  Clinician roles: 46 doctors, 24 nurses, 27 allied health, 10 administrators  Analyzed using uncertainty index (% of staff with no clear attitude), intensity index (% of staff with strong view – either strongly agree or strongly disagree), polarity index (spread of group attitudes – high polarity is ~equal % of respondents agreeing and disagreeing with an item), overall positivism to CDs (answers to 20 items focusing on CDs in general) | Outcome category: Work attitudes  Staff attitudes toward:  Clinician issues (e.g. decision making, autonomy): slightly polarized, 26% of respondents were uncertain, 25% held intense attitudes  Working relationships: most polarized section – wide disagreement here, but low intensity of responses (12%); uncertainty was high (37%)  Coordination and management issues: attitudes were in mid-range of polarity, uncertainty, and intensity  Decentralization: all scores in mid-range  Organizational performance and benefits: medium polarity, low intensity, and high uncertainty  Comparison of managers and non-managers: managers were less uncertain, less polarized, and more intense in their responses  Results suggest staff were uncertain about governing arrangements of the hospital, its purpose, contribution, and effects  No patient outcomes reported |
| Castle, 2006 USA [77] Quality rating: 10.5/17 | | | | |
| Structure of nursing homes (for profit status and membership in nursing home chain) | Nursing (registered nurses [RN], licensed practical nurses [LPN], certified nurse aides [CNA]) | Turnover  Authors’ conceptual model has   1. chain membership 🡪 autonomy 🡪 job satisfaction 🡪 turnover 2. profit status 🡪 pay and benefits 🡪 job satisfaction 🡪 turnover 3. top management turnover (proxy for leadership) 🡪 institutional loyalty 🡪 job satisfaction 🡪 turnover   Turnover is the only outcome measured  Also discussed workload, professional interactions, institutional loyalty, relationships, job pride, but not in relation to governance factors | Data from Online Survey, Certification, and Reporting (OSCAR), Area Resource File, survey of nursing home administrators  N= 854 facilities | Outcome category: Retention  No relation between chain membership and turnover  No relationship between management turnover (proxy for leadership) and LPN or all staff turnover; relationship between management turnover and CNA and RN turnover  Not-for-profit nursing homes were associated with lower nursing staff turnover (for CNA, LPN, RN, and all combined)  No patient outcomes reported  Similar study to Donoghue 2009 but datasets appear to be from different years |
| Donoghue, 2009 USA [76] Quality rating: 12/17 | | | | |
| Structure of nursing homes (for profit status and membership in nursing home chain) | Nursing (RN, LPN, Nursing Aide [NA]) | Turnover  Model uses job satisfaction as partial mediator between organizational/leadership factors and turnover, also proposes direct effects: low job satisfaction 🡪 turnover  Also suggested that staff needs to be empowered to make decisions about patient care, which would also lead to higher job satisfaction | Data from National Nursing Home Turnover Study (nationally representative survey) and OSCAR database (data from state and federal nursing home inspections)  N = 2900 nursing homes | Outcome category: Retention  For profit status associated with lower turnover in LPNs, not predictive for RNs or NAs  Chain status associated with higher turnover in RNs and LPNs, no effect on NAs  Other human resource factors examined, but only as predictors of turnover  No patient outcomes reported  Similar study to Castle 2006 but datasets appear to be from different years |
| Lavoie-Tremblay, 2011 Canada [71] Quality rating: 14/17 | | | | |
| Quebec Ministry of Health’s Mental Health Action plan and resulting organizational models in two hospitals; models based on patient-centred care; favours public access to care, quality of life, effectiveness and efficiency of healthcare system, and hierarchization of care, informed by evidence | Psychiatrists, nurses, psychologists, OT, social work | Psychological distress, job strain, recognition (effort/reward imbalance), use of evidence  Also mentioned: patient-centred care gives providers the necessary latitude to plan and perform their work to provide the best response to the patient’s needs and improve the processes resulting in increased job satisfaction; however, some research suggests it also results in greater anxiety and more questions from providers regarding care and job security  Also discussed psychological demands (amount and complexity of work, job constraints) and decision latitude, social support – suggests social support from colleagues and superiors should moderate effects of job strain; Effort-Reward Imbalance model suggests that work situation with high degree of effort expended combined with little reward received can have pathological effects on health of employees | Case 1: Department of psychiatry at academic hospital; 24 respondents completed questionnaire Time 0 and Time 2  Case 2: Mental health outpatient services at psychiatric hospital; 38 respondents completed both times  Self-report questionnaires administered at one-year intervals (time 0, 1, 2) and focus groups with semi-structured interview | Outcome category: Work attitudes, care protocols  Case 1:  Quantitative: No significant changes in effort/reward ratio, social support from colleagues, social support from superiors, psychological demand, or use of evidence; decrease in decision latitude and increase in psychological distress  Case 2:  Quantitative: No significant changes in effort/reward ratio, decision latitude, psychological demand, or use of evidence; increase in social support from colleagues and psychological distress, decrease in social support for superiors  Qualitative: Patient-focused care allows interdisciplinary teamwork, this is rewarding; more flexibility in terms of practice, room for creativity; workload remains heavy because few links with frontline to transfer patients; changes to training and work organization are psychologically demanding  No patient outcomes reported |
| McCloskey, 2005 New Zealand [90] Quality rating: 11/17 | | | | |
| Reengineering by New Zealand (NZ) government – included creation of healthcare market (expected increased competition among providers once government was no longer sole provider and purchaser; never fully developed), replacement of traditional leadership with business managers, managerialism | Nursing (RNs and enrolled nurses [ENs; similar to LPNs]) | Skill mix (percentage of total nursing full time equivalents [FTEs] who were RNs)  Change to FTEs, change to hours worked | Retrospective analysis of longitudinal administrative data using time series design, from 1993 to 2000, to examine effects of reengineering policies on adverse patient outcomes and the nursing workforce  Two databases run by NZ Health Information Service: National Minimum Dataset (NMDS) and Nursing Workforce Dataset (NWD)  NMDS: Patient level discharge abstracts  NWD: Completion is mandatory; study is based on sample of 65,221 nurses | Outcome category: Skill mix, workload  Skill mix increased 18% from 1993 to 2000  36% decrease in RN and EN FTEs, 36% decrease in hours worked per 1000 discharges; FTEs and hours worked by 1000 patient days deceased by 9% each (increased workload result of 70% decrease in number of ENs)  Skill mix result was probably because of phasing out EN role in hospitals, rather than higher mix of RNs actively being sought  Patient outcomes: Preventable adverse outcomes, based on 11 nurse sensitive clinical outcomes (NSCO)  Increases in 7 NSCOs  No change in remaining 4 NSCOs  Average length of stay decreased  Mortality for medical discharges decreased, remained stable for surgical discharges  Patient outcomes and nursing workforce: Statistically significant relationships between several adverse outcome rates and each of the following: decreases in number of nurses in hospital workforce, decreases in number of nursing hours worked, and increases in skill mix |
| O’Dowd, 2006 UK (Ireland) [73] Quality rating: 10/17 | | | | |
| Two out-of-hours cooperatives for general practice (GP) physician services in rural and mixed urban/rural areas  Differences between cooperatives:  – one call centre used nurses to triage patients the other used GPs  – one with paid hourly rate, the other with pay on consultation basis (no monetary reimbursement for phone consults, general medical services consults or down-time, i.e. no patients waiting to be seen) | Physicians | Quality of life, satisfaction with various aspects of the cooperative (cooperative responsibilities, shifts, complaint process, other staff, confidence for out-of-hours work, supplies, independence, pay, decision making)  Discusses implications for: Continuing education needs (mental health, palliative), potential role of other providers (nurses) and availability of support services (dental, mental health, social services) | Questionnaire sent to all GP members of two cooperatives (82% responded, n = 182) | Outcome category: Work attitudes  Improvements in their own quality of life (97%), quality of family/social life (91%), ability to cope with demands of work (75%), stress levels (77%) as a result of joining cooperative  Half of providers reported feeling overburdened by cooperative responsibilities (slightly greater proportion of those aged 40 yrs or more)  Dissatisfaction with number of shifts after midnight (20%), advance notice of shifts (16%), frequency of shifts worked (12%), procedures to deal with own complaints (almost 1/4)  Satisfaction with medical and support staff (94%), method by which shifts are allocated (92%), own confidence for out-of-hours work (95%), provision of medicines and equipment (90%), independence in deciding how to treat patient (96%)  Differences between cooperatives:  Satisfaction with amount paid similar (46% in consultation, 41% in hourly), but satisfaction with method of payment higher in hourly (84%) than in consultation (58%)  63% prefer health board-GP partnership be responsible for organization of out-of-hours care vs. 23% who prefer GP responsibility alone [speaks to governance of the cooperative]  No patient outcomes reported |
| Sicotte, 2002 Canada [72] Quality rating: 15/17 | | | | |
| Quebec Centres Locaux de services communautaires (CLSCs)/ Community Health Care Centres (CHCC) | Program coordinators | Interdisciplinary collaboration  Also mentioned: professional autonomy and jurisdiction (vs. collaborative behaviour), open communication, conflict resolution, managing social status group differences  Model: contextual factors 🡪 intragroup factors 🡪 intensity of interdisciplinary collaboration  Collaboration is mediated by nature of the task | 343 questionnaires from program coordinators (elderly home care, youth and family care, ambulatory walk-in clinics, specialized adult care) from 157 CHCCs  Measured intensity of interdisciplinary collaboration (care sharing activities and interdisciplinary coordination); determinants of interdisciplinary collaboration (contextual variables: characteristics of program managers, structural characteristics of the program, e.g. formalization of care activity procedures and assessment of quality of care; intragroup processes: beliefs in benefits of collaboration, social integration within groups, level of conflicts, agreement with disciplinary logic, agreement with interdisciplinary logic); work group design characteristics | Outcome category: Collaborative practice  Intensity of interdisciplinary collaboration is moderately positive (over 3.5/5 for all programs); authors note this is disappointingly low given stated goal of CHCCs is collaboration  Regression results show that contextual variables are not associated with the intensity of interdisciplinary collaboration  Neither the organizational characteristics of the programs nor the characteristics of the program coordinators are statistically significant predictors of interdisciplinary collaboration; contextual variable associated with intensity of collaboration is ‘‘formalization of the assessment of quality of care’’ (a structural characteristic of the programs that is statistically associated with both measures of collaboration intensity)  Intragroup process variables (beliefs in benefits of collaboration, social integration within groups, level of conflicts) explain most of the variance in intensity of interdisciplinary collaboration  Main factors associated with interdisciplinary collaboration are closely linked to work group internal dynamics  No patient outcomes reported |
| Silvestro, 2008 UK [74] Quality rating: 11.5/17 | | | | |
| Rostering (i.e. nurse scheduling) practices in hospitals in the UK | Nursing | Recruitment, morale, absenteeism, retention, skill mix | Telephone survey of nurse managers to identify parameters of rostering systems and document factors taken into account in designing unit rosters  Examination of over 50 sample rosters  Longitudinal case studies of five wards in two hospitals | Outcome category: Work attitudes, recruitment, retention, skill mix, absenteeism  Objectives common to all roster-planning activities: manning requirements (maintain appropriate patient care, while not over-working staff or under-utilizing their time); cost control (minimizing use of casual staff); staff requirements (shift allocations, meeting expectations for flexible working, ensuring equitable treatment – staff may be absent if assigned bad shifts), tacit objectives (e.g. need to maximize staff pay by working undesirable hours)  Need to account for hospital recruitment and retention strategies, legal requirements (e.g. European working time directive), national retention strategies, required skill mix  Possible outcomes from poor design (relevant only): under-manning the ward, compromising patient care and increasing staff stress levels; inability of staff to integrate their home/professional lives; low morale and poor relations between management and staff; absenteeism, particularly if the ward was understaffed and therefore a stressful place to work; high staff turnover; poor employer reputation, making it difficult to attract new staff; failure to realize wider national and hospital strategies to improve retention such as the Improving Working Lives Initiative and the European Working Time Directive  No patient outcomes reported |
